# Temperature-regulated guest admission and release in microporous materials

**DOI:** 10.1038/ncomms15777

**Published:** 2017-06-09

**Authors:** Gang (Kevin) Li, Jin Shang, Qinfen Gu, Rohan V. Awati, Nathan Jensen, Andrew Grant, Xueying Zhang, David S. Sholl, Jefferson Z. Liu, Paul A. Webley, Eric F. May

**Affiliations:** 1 Centre for Energy, School of Mechanical & Chemical Engineering, The University of Western Australia, 35 Stirling Highway, Crawley, Western Australia 6009, Australia; 2 Joint Laboratory for Energy and Environmental Catalysis, School of Energy and Environment, City University of Hong Kong, Tat Chee Avenue, Kowloon, Hong Kong SAR, People's Republic of China; 3 Department of Chemical and Biomolecular Engineering, The University of Melbourne, Melbourne, Victoria 3010, Australia; 4 Australian Synchrotron, 800 Blackburn Rd, Clayton, Victoria 3168, Australia; 5 School of Chemical & Biomolecular Engineering, Georgia Institute of Technology, Atlanta, Georgia 30332-0100, USA; 6 Department of Mechanical and Aerospace Engineering, Monash University, Clayton, Victoria 3800, Australia

## Abstract

While it has long been known that some highly adsorbing microporous materials suddenly become inaccessible to guest molecules below certain temperatures, previous attempts to explain this phenomenon have failed. Here we show that this anomalous sorption behaviour is a temperature-regulated guest admission process, where the pore-keeping group's thermal fluctuations are influenced by interactions with guest molecules. A physical model is presented to explain the atomic-level chemistry and structure of these thermally regulated micropores, which is crucial to systematic engineering of new functional materials such as tunable molecular sieves, gated membranes and controlled-release nanocontainers. The model was validated experimentally with H_2_, N_2_, Ar and CH_4_ on three classes of microporous materials: trapdoor zeolites, supramolecular host calixarenes and metal-organic frameworks. We demonstrate how temperature can be exploited to achieve appreciable hydrogen and methane storage in such materials without sustained pressure. These findings also open new avenues for gas sensing and isotope separation.

## Abstract (web summary)

Regulating guest access and release in porous materials remains an important goal. Here, May and colleagues elucidate the mechanism by which guest admission can be temperature-regulated in typical microporous materials, and experimentally exploit this process to achieve appreciable and reversible hydrogen storage.

Selective admission of guest molecules into microporous solids is the cornerstone of a range of key applications such as gas separation^1^,^2^,^3^,^4^,^5^, storage^6^,^7^ and recognition^8^,^9^. However, in many important cases the size difference between relevant molecules can be of order 0.1 Å (refs 10, 11), and materials with the appropriately sized micropores necessary for discrimination are often very difficult to find^12^. If the accessibility of the micropores could be actively regulated by external stimuli, such as heat^13^, light^14^ or electrical field, the material's utility could be fundamentally enhanced. In 1959, Breck showed that at temperatures below 170 K, the admission of Ar and N_2_ molecules into 4A zeolite was dramatically reduced^15^. Recently, other microporous materials including molecular trapdoor zeolites^3^,^16^,^17^ and certain metal-organic frameworks (MOFs)^13^,^18^,^19^,^20^,^21^,^22^ have been reported to exhibit similar properties, with negligible N_2_ surface area at 77 K but considerable adsorption at ambient temperatures. Many other zeolites also exhibit such a pore-blocking effect towards inert gases including caesium chabazites^23^, RHO^24^,^25^ and K-KFI^26^. While observations of this phenomenon imply pore accessibility can be regulated by temperature, neither the underlying science nor the process by which this regulation occurs is fully understood.

To elucidate a mechanism by which the admission of guest molecules can be thermally regulated, this work presents experimental data and *ab initio* calculations that explain the phenomenon for several types of microporous materials including trapdoor zeolites, supramolecular hosts^27^,^28^ and MOFs. Furthermore, a physical model is proposed to provide a quantitative description of temperature-regulated guest admission. Finally, we demonstrate a new application of this temperature-regulated gas admission phenomenon in which appreciable storage of H_2_ can be achieved without sustained external pressure via encapsulation within the material's pore space. Importantly, the amount of hydrogen encapsulated greatly exceeds the equilibrium adsorption capacity of the material at comparable conditions.

## Results

### Experimental study of temperature-regulated guest admission

**Figure 1:**
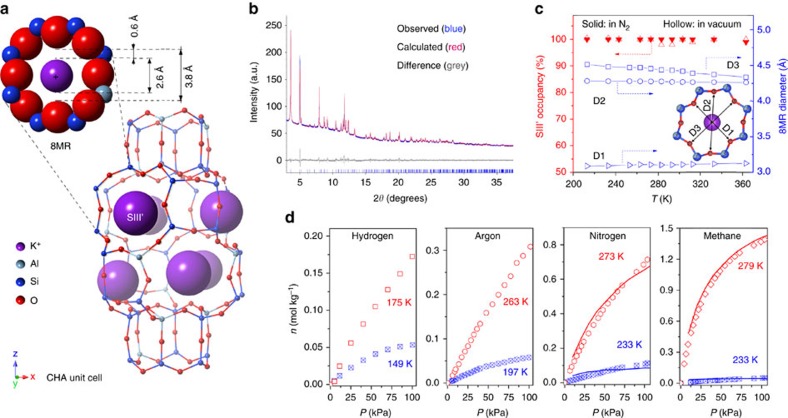
Paradox of non-polar gas molecule admission through the apparently blocked pores of a potassium chabazite. (**a**) Illustration of the structure of an ideal potassium chabazite crystal, highlighting the eight-membered oxygen ring (8MR) which sets the nominal pore aperture diameter to be 3.8 Å (distance between opposing oxygen atoms). The centre of the 8MR pore aperture (cation site SIII′) is occupied by the potassium cation, reducing the nominal accessible pore aperture to 0.6 Å, as determined from the Synchrotron PXRD data. (**b**) Rietveld refinement of a representative Synchrotron PXRD pattern for trapdoor potassium chabazite with a Si/Al ratio of 2.2 (**r2**KCHA). (**c**) Left axis: fractional occupancy of SIII′ sites by K^+^ cations indicating blockage of 8MR pores over the experimental temperature range. Estimated uncertainty 1.5%. Right axis: evolution of the 8MR dimensions D1, D2 and D3 (inset) of **r2**KCHA suggesting pore contraction with increasing temperature, ruling out the possibility of thermally induced pore dilation. 0.3% uncertainty. (**d**) Adsorption isotherms for hydrogen, argon, nitrogen and methane, respectively, on **r2**KCHA showing restricted pore accessibility at low temperatures (crossed blue symbols) but no restriction at high temperatures (red open symbols), and comparison with GCMC calculated equilibrium capacities (lines) for nitrogen and methane.

To exemplify the phenomenon of temperature-regulated guest admission, we show in Fig. 1 how pore accessibility can be abruptly and reversibly switched on/off across a narrow temperature range for four different guest molecules on a trapdoor^16^ chabazite, **r2**KCHA (potassium chabazite with Si/Al ratio of 2.2). This material is a typical small-pore zeolite in which eight-membered oxygen rings (8MRs) govern the access of guest molecules to internal cavities (Supplementary Figs 1 and 2). Geometrically, the 8MR is large enough for simple gas molecules such as H_2_ and Ar to pass through, but in trapdoor zeolites like **r2**KCHA, this access is blocked by large cations which occupy the energetically favourable site (SIII′) at the centre of the 8MR doorway^16^,^17^, as exemplified by a low N_2_ surface area at 77 K, which for **r2**KCHA was 21 m^2^ g^−1^. A sharp transition in the pore accessibility with temperature is clearly apparent for **r2**KCHA in the uptake of H_2_, Ar, N_2_ and CH_4_ (Fig. 1d). The ultra-low adsorption capacities observed for these molecules at 149, 197, 233 and 233 K, respectively, transition to considerable capacities with an increase of only 30–70 K. This is contrary to the normal dependence of physisorption capacity on temperature^29^,^30^, as illustrated in Supplementary Fig. 3.

One popular hypothesis for temperature-dependent pore accessibility is thermal expansion of the pore size^31^,^32^. We excluded this possibility by *in situ* synchrotron powder X-ray diffraction (PXRD) measurements of **r2**KCHA (Fig. 1b,c) over the temperature range of 213–363 K under vacuum and N_2_, respectively. The Rietveld refined PXRD data as detailed in Supplementary Note 1 and Supplementary Tables 1 and 2 show that while the accessible opening remained less than 1 Å, the 8MR diameter actually contracted slightly by 0.18 Å with increasing temperature. Such negligible negative thermal expansion is not uncommon in zeolites or other microporous materials^33^,^34^. Similarly, potential explanations based on restricted diffusion (kinetic) effects were excluded through adsorption rate measurements (detailed in Supplementary Fig. 4 and Supplementary Note 2).

Another possible mechanism is the migration of the pore-keeping cation from the 8MR centre to a secondary stable site at sufficiently high temperatures above a threshold, as suggested by Shang *et al*.^16^ for a caesium-exchanged chabazite. However, the PXRD data, which also resolved the locations of the extra-framework cations, eliminate this explanation in the case of **r2**KCHA. Figure 1c shows that the favourable cation site SIII′ at the centre of the 8MR doorways remains fully occupied by K^+^ across the entire temperature range. Superficially this temperature-independent cation position suggests that the admission of N_2_ into the **r2**KCHA pores should remain negligible at least up to 363 K. The question then arises how H_2_, Ar, N_2_ and CH_4_ were admitted while the pore entrance was seemingly blocked.

### *Ab initio* DFT studies of gas admission

We explored whether guest admission in **r2**KCHA is regulated by the temporary and reversible deviation of the pore-keeping K^+^ cation from the SIII′ site by calculating the associated potential energy surfaces using *ab initio* DFT (density functional theory). The admission process was simulated as consisting of two concurrent parts: the pore-keeping K^+^ cation deviates temporarily from the 8MR's centre (SIII′) as a result of an interaction with the guest molecule, which allows the guest molecule to pass through the transient opening in the 8MR doorway. Accordingly, the energy barrier (Δ*E*_total_) of this coupled gas admission process contains two interdependent components: Δ*E*_total_=Δ*E*_host_+Δ*E*_guest_, where Δ*E*_host_ accounts for the potential energy change of the cation's temporary deviation in the presence of a gas, and Δ*E*_guest_ is the potential energy barrier a guest molecule passing through the temporarily opened doorway must overcome, due to the cation and framework. We used an iterative method within the DFT calculations to capture the energy profiles associated with the simultaneous movement of the cation and the gas molecule (Supplementary Note 3).

**Figure 2:**
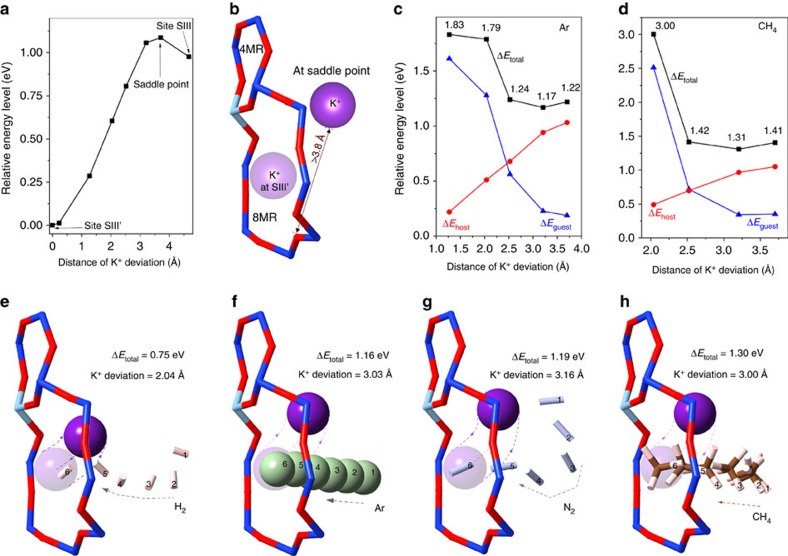
Atomic picture of the guest admission process through the cation-kept pores in potassium chabazite. (**a**) Energy profile for the deviation of the potassium cation following the SIII′-to-SIII path. (**b**) The position of the saddle point relative to the original cation site SIII′, which if reached would completely unblock the 8MR doorway to incoming guests. (**c**,**d**) Examples of DFT-calculated energy barriers experienced by the door-keeping cation of the host zeolite, the interacting guest molecule (**c**, argon and **d**, methane, respectively) and their sum as a function of cation position. With the increasing deviation there is a trade-off between the increase in the cation's potential energy and a decrease in the potential energy barrier experienced by the guest molecule. (**e**–**h**) Total energy barriers and trajectories for the admission of guest molecules H_2_, Ar, N_2_ and CH_4_, respectively. Higher energy barriers must be overcome for the admission of larger guest molecules; however, larger molecules do not necessarily require the cation's deviation to be greater in extent because there are two (guest and host) contributions to the total energy barrier.

We identified that the transient deviation of the K^+^ cation follows the pathway from SIII′ to SIII (Fig. 2), that is, from the centre of an 8MR to the site above the adjacent 4MR plane (see Supplementary Figs 5–7 for detailed justification). As illustrated in Fig. 2a,b, if the cation deviated temporarily to the potential energy saddle point along the SIII′-to-SIII path, these guest molecules would have more than enough room to pass through the 8MR doorway; that is, the cation does not need to reach the secondary stable site SIII to admit guest molecules. By mapping Δ*E*_total_ in the presence of different guest molecules as a function of cation position along the SIII′-to-SIII pathway, we determined the minimum energy required for the admission of each gas. In all cases the minimum Δ*E*_total_ of the system was achieved before the cation deviation reached the saddle point in Fig. 2a,b. Examples for Ar and CH_4_ are shown in Fig. 2c,d, and Δ*E*_total_ for the four gases follows the order H_2_ (0.75 eV) <Ar (1.16 eV) <N_2_ (1.19 eV) <CH_4_ (1.30 eV). The trajectories determined by DFT calculations (Fig. 2e–h) clearly show that a partially opened 8MR doorway is sufficient for the admission of these molecules. However, they also illustrate that the deviation distance of the cation, that is, the opening of the doorway, is not necessarily proportional to the size of the guest molecule (Fig. 2g,h), indicating that this guest admission process is not simply steric in nature.

### GCMC calculated adsorption equilibrium

We conducted Grand Canonical Monte Carlo (GCMC) simulations to compute the equilibrium adsorption isotherms for CH_4_ and N_2_ on **r2**KCHA at all temperatures, using force field parameters derived from both experiment and literature^35^,^36^. A 3 × 3 × 3 supercell was built to take into account the temperature-dependent pore accessibility (Supplementary Figs 8 and 9). Good agreement between the GCMC-calculated and experimentally measured adsorption capacities was achieved across the whole temperature range (Supplementary Figs 10 and 11), including during the transition from accessible to inaccessible pores with decreasing temperature. More details about the determination of the simulation parameters are presented in Supplementary Note 4 and summarized in Supplementary Tables 3–6 (refs 37, 38).

### A quantitative model for regulated guest admission

To derive a physically based function able to describe the observed temperature dependence of the guest admission process, we start with a comparison between the minimum energy barrier Δ*E*_total_ and the thermal energy of the system. The system's thermal energy *E*_therm_ is the sum of the kinetic energy of the guest molecule interacting with the pore-keeping groups and the thermal vibrational energy of those groups in the framework of the regulated pores. A general expression for the mean thermal energy *Ē*_therm_ is (ref. 39):


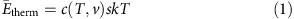


where *s* is the number of harmonic oscillators (or degrees of freedom) of the system, *k* is the Boltzmann constant and *c*(*T*, *v*) is a function of the absolute temperature *T* and the oscillators' vibrational frequencies *v*. In systems of harmonic oscillators where the number of degrees of freedom is large (for example, 10^2^–10^3^ Da as for the pore-structures considered here) the energy distribution will be Gaussian^39^,^40^. Accordingly, the probability density distribution of their thermal energies, *E*_therm_, is:


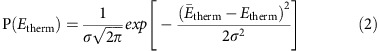


Here *σ* is the standard deviation of the distribution function, representing its width in terms of energy. The cumulative probability that a given oscillator will admit a guest molecule is then:


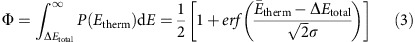


Identifying the threshold temperature, *T*_0_, at which the system's mean thermal energy is equal to Δ*E*_total_, and substituting the expression for *Ē*_therm_ from equation (1) into equation (3) gives:


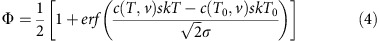


Drahos and Vékey suggest that below 1,000 K, both *c*(*T*, *υ*) and *σ* can be well approximated by linear functions of temperature^40^. Accordingly, the final analytical expression for the fraction of pore-keeping oscillators that allow guest molecule admission as a function of temperature is:


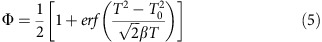


where *β* characterizes the width of the system-specific distribution in temperature. The product of *βT* in equation (5) is the standard deviation of the cumulative distribution function, indicating that the width of the distribution is asymmetrical and broader at higher temperatures (Supplementary Fig. 12).

This temperature-dependent distribution function for the accessibility of internal sites can be readily incorporated into a classical model of equilibrium adsorption capacity *Q*(*P*, *T*), such as Langmuir, Sips, Toth or BET^30^, to enable a quantitative description of temperature-regulated guest admission:


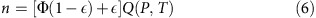


Here *Q*(*P*, *T*) is the classical isotherm as a function of temperature and pressure. The pre-factor in square brackets contains the fraction of accessible adsorption sites determined by the temperature-dependent distribution function, Φ, together with the fraction of external surface sites and defects always available for adsorption, *ɛ*. For near-perfect adsorbent crystals with sufficiently large grain size *ɛ* will be negligible. Alternatively *ɛ* can be estimated through surface area analysis with specific gases. The fraction Φ can also be found from GCMC calculations.

We name the above method of describing the stimuli-dependent availability of adsorption sites as the LJM model, and accordingly LJM-Toth and LJM-Langmuir denote models (Supplementary equations 2 and 3) for heterogeneous and homogeneous adsorption, respectively. The LJM model introduces only two new parameters, namely *T*_0_ and *β*, to describe the temperature dependence of the pore accessibility. All other parameters describing the adsorption occur within the classical isotherm equations. To determine *T*_0_ and *β* reliably, equation (6) should be regressed to adsorption isotherms measured over a sufficiently wide temperature range spanning the material's transition from inaccessible to accessible pores. The physical significance of *T*_0_ and *β* was further established when it was found that including Φ into the GCMC calculations enabled accurate prediction of adsorption capacities measured in the transitional region. In principle, LJM models are compatible with ideal adsorbed solution theory^41^ and other multicomponent isotherm models (Supplementary Note 5) and can also be used to calculate the isosteric heat of adsorption (Supplementary Fig. 13)^42^,^43^.

### Demonstration for trapdoor zeolites

**Figure 3:**
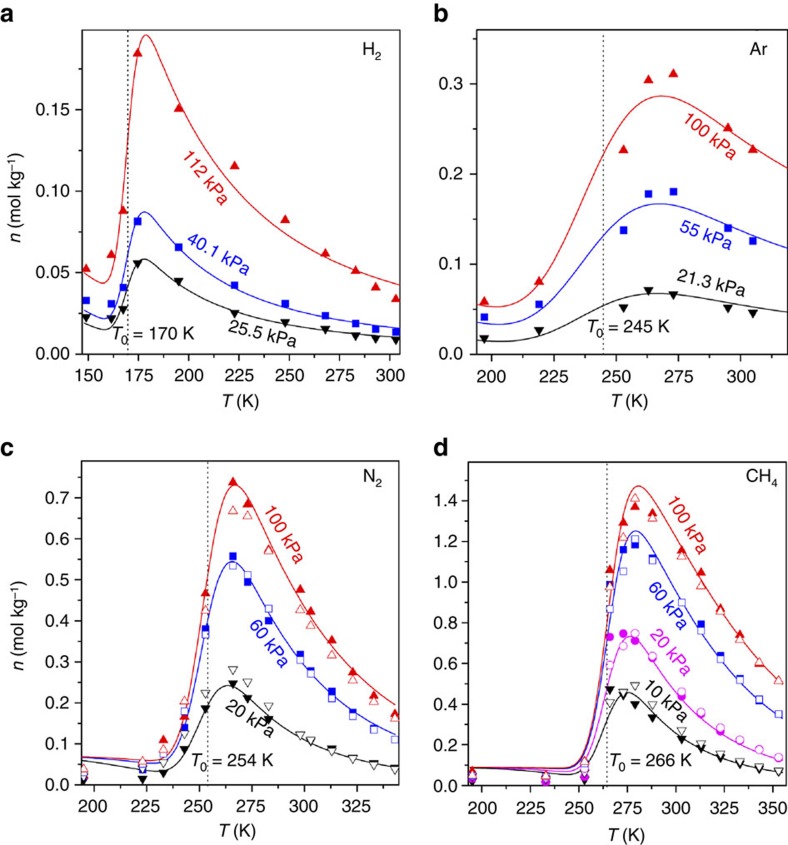
Representative adsorption isobars for four gases on the potassium chabazite. Experimentally measured data together with the GCMC calculations and a demonstration of the LJM model's ability to quantify the effect of temperature-regulated guest admission. (**a**) H_2_ at 11 different temperatures; (**b**) Ar at 7 temperatures; (**c**) N_2_ at 14 temperatures; (**d**) CH_4_ at 13 temperatures. Only 1/6^th^ of the isobar data points measured and used in the regression are shown here for clarity. Full isotherm/isobar data across the experimental pressure range 2–120 kPa can be found in Supplementary Figs 14–17. The full set of N_2_ and CH_4_ GCMC calculations are presented in Supplementary Figs 10 and 11. Solid symbols denote experimental data, hollow symbols represent the GCMC calculated capacities, and solid curves correspond to the LJM-Langmuir model. The dotted lines represent the threshold admission temperature *T*_0_ determined using the LJM model.

The LJM model was used to describe successfully the temperature-regulated admission of several non-polar guest species on three classes of microporous materials across ranges of at least 108 K. First, adsorption isotherms were measured for H_2_, Ar, N_2_ and CH_4_ on the molecular trapdoor zeolite **r2**KCHA, and regressed by the LJM-Langmuir model (Fig. 3). The experimental, GCMC and LJM capacities are all in good agreement across the pore-accessible, transitional and pore-inaccessible regions. The quality of the fits to the data is high: for example, measurements of the **r2**KCHA—N_2_ system spanned 14 isotherms (195–343 K) over the pressure range of 1–120 kPa with 312 data points in total. Description of the eight N_2_ isotherms below 273 K would be impossible without the two LJM parameters. The regression had an *R*^2^=0.992 with all parameter values being physically meaningful. Similar results were obtained for the other three gases as shown in Supplementary Table 7.

**Figure 4:**
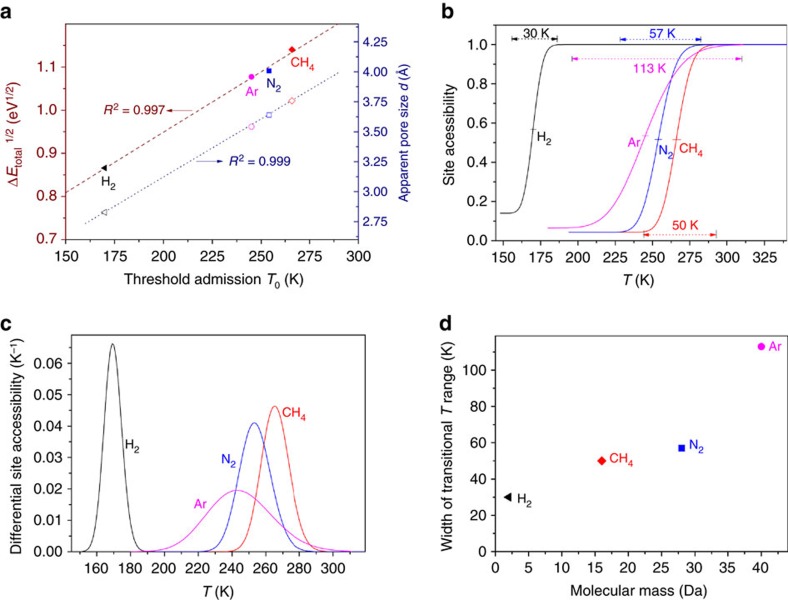
Physical significance of the temperature-regulated guest admission model parameters. (**a**) Left axis: Linear correlation between the square root of the total energy barrier Δ*E*_total_ and the threshold guest admission temperature *T*_0_, indicating a consistency between the DFT calculations, framework used to derive the LJM model, and the experimental data. Right axis: linear relationship between *T*_0_ and the apparent pore size of **r2**KCHA based on the guest molecule's kinetic diameter^10^,^12^. (**b**) Accessibility of adsorption sites as a function of temperature for guest molecules of H_2_, CH_4_, N_2_ and Ar as calculated by 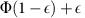 in equation (6). The lower and upper cut-offs are 0.1 and 99.9% of the accessibility of the internal sites, respectively, and the initial value of the curve represents the fraction of external sites *ɛ*. (**c**) Corresponding differential site accessibility for the four different guest molecules on **r2**KCHA. (**d**) Corresponding width of the transitional temperature range (as characterized by *β* in the LJM model) for the admission of different guest species, showing heavier molecules tend to have larger distribution widths.

Correlating the experimentally determined *T*_0_ values with the DFT calculated energy barriers Δ*E*_total_ for the admission of H_2_, Ar, N_2_ and CH_4_ on **r2**KCHA produced a striking relationship. As shown in Fig. 4a, *T*_0_ is linearly proportional to the square root of Δ*E*_total_. This relationship is consistent with the use of equations (1), (3) and (4) in the derivation of the LJM model and supports the interpretation of *T*_0_ as the temperature at which the mean thermal energy of the adsorption system reaches a threshold value sufficient to overcome the guest admission energy barrier Δ*E*_total_. Furthermore, the experimental *T*_0_ are proportional to the kinetic diameters^10^,^12^ *d* of the guest molecules: in the context of the mechanism elucidated by the DFT results, this property will facilitate the design of tuneable molecular sieves and thermally gated membranes from zeolites. A third interesting correlation observed in the experimental data (Fig. 4b–d) relates to the order of the distribution width *β* and the molecular mass of each guest species: H_2_ (30 K), CH_4_ (50 K), N_2_ (57 K) and Ar (118 K). This result has important implications for the potential use of temperature-regulated sieving to separate isotopes (for example, hydrogen-deuterium-tritium^44^).

### Demonstration for calixarenes and MOFs

**Figure 5:**
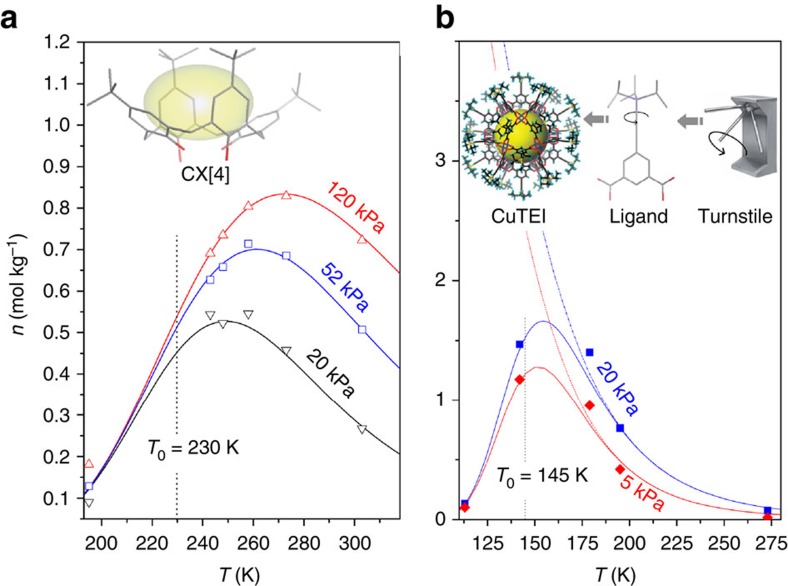
Demonstration of temperature regulated guest admission on supramolecular hosts and MOFs. (**a**) Typical bell-shaped isobars for the adsorption of CH_4_ on *p*-*t*-butylcalix[4]arene showing good agreement between the experimental data (points) and the LJM model (solid curves). A threshold admission temperature of 230 K was determined by the model's regression. Inset: molecular structure of *p*-*t*-butylcalix[4]arene showing the accessibility of its basket-shaped cavity is kept by four thermally responsive *t*-butyl groups. (**b**) Representative adsorption isobars for CH_4_ on CuTEI, a MOF nanocage whose ligand 5-((triisopropylsilyl)ethynyl)isophthalic acid (TEI) provides a turnstile-type configuration. Symbols denote experimental data, solid curves the fitting by LJM-Toth model, and dashed curves the inevitable overestimation of the guest uptake using conventional physisorption models. Inset: illustration of the turnstile type pore keeping group in CuTEI (the cage structure of the CuTEI was adapted from Fig. 1 in *Chem. Commun*. **46,** 7352–7354 (2010) with permission from The Royal Society of Chemistry). For clarity, less than 1/5^th^ of the data points used in the regression are presented in **a**,**b**. The full data set is available in the Supplementary Table 8.

Calixarenes are typical supramolecular host materials composed of macro-cyclic polyphenols. The cyclic tetramer calix[4]arene has a stable basket conformation and is known to accommodate small guest molecules, such as CH_4_ and N_2_ (refs 27, 28). Its host–guest inclusion complex formation has similar characteristics to physisorption. In this study, methane adsorption isotherms were measured on *p*-*t*-butylcalix[4]arene between 195 and 303 K at pressures up to 120 kPa (Supplementary Fig. 18). The data were regressed successfully to the LJM-Langmuir model (Fig. 5a), revealing a threshold temperature of 230 K for CH_4_ admission. The results can be interpreted in terms of the cooperative rotation of the *tert*-butyl groups producing a ‘turnstile' effect which regulates the admission of guests into the CX[4] cavity^45^.

Adsorption data reported for a number of MOFs also exhibit the bell-shaped isobars characteristic of the temperature-dependent gas admission effect^13^,^20^,^22^,^46^. Interestingly, all these MOFs have pore-keeping groups responsive to temperature changes (Supplementary Fig. 19). For example, adsorption capacity data have been presented for a porous coordination nanocage, CuTEI^13^, the exterior of which is covered by turnstile-like triisopropylsilyl groups that regulate access to the nanocage. Regression of the LJM-Toth model to the 93 literature data collected between 113 and 273 K produced a reasonable fit (Fig. 5b), with *T*_0_=145 K, suggesting a much smaller energy barrier for CH_4_ admission compared with CX[4]. This result highlights the importance of examining the possibility of temperature-dependent pore accessibility changes in ‘designer' microporous materials, especially those generated and screened purely by computer^13^,^47^, to avoid substantial over-estimation of lower-temperature adsorption capacities.

### Reversible hydrogen and methane encapsulation and release

**Figure 6:**
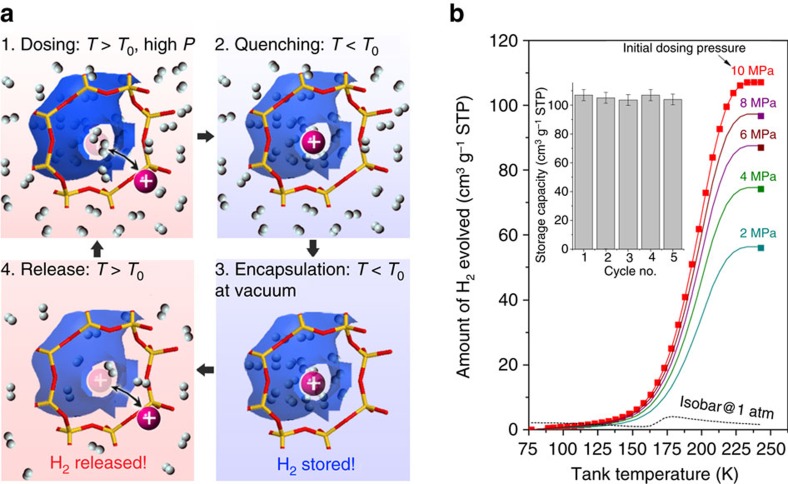
Physical storage of H_2_ by encapsulation without sustained external pressure. (**a**) Four-step procedure for reversible encapsulation and decapsulation of H_2_ in materials with temperature-regulated guest admission: (1) At temperatures above the threshold, *T*_0_, high-pressure dosing is used to store a significant quantity of H_2_ within the material's pore volume. (2) The material is quenched to below *T*_0_ so the door-keeping group closes off access between the pore space and the environment. (3) H_2_ remains encapsulated inside the material's pore space even when the external pressure is replaced with a vacuum as long as the temperature is below *T*_0_. (4) Controlled decapsulation of the gas into the low pressure environment is achieved by bringing the system temperature above *T*_0_, with the cumulative amount evolved providing a measure of the initial storage. This storage and release cycle is fully reversible and regulated by heat transfer. (**b**) Measured amount of H_2_ decapsulated from **r2**KCHA during step (4) as a function of system re-heating temperature after dosing with different initial pressures. Inset: cyclical performance of H_2_ encapsulated at a dosing pressure of 10 MPa by following the above four-step procedure across five consecutive experiments, showing steady and repeatable H_2_ storage capacity (see Supplementary Table 9 for detailed data).

The storage of H_2_ and CH_4_ in porous media such as MOFs and carbons with large surface areas is a widely studied approach for achieving high volumetric energy density with relatively low regeneration cost. Common drawbacks of such approaches include the unavoidable use of thick-walled vessels to contain the high pressure and/or limited reversibility of the storage process^6^,^48^. The temperature-regulated guest admission mechanism identified here offers substantial potential for such applications because pre-dosed gas can be encapsulated and prevented from escaping by lowering the system temperature below *T*_0_. We explored this concept (Fig. 6a) by dosing the **r2**KCHA trapdoor zeolite at ambient temperature with H_2_ at various pressures up to 10 MPa using a set up shown in Supplementary Fig. 20. After quenching to cryogenic temperatures, the system was evacuated to remove all the free gaseous hydrogen, leaving only the hydrogen trapped inside the chabazite's intracrystal cavities. No H_2_ was evolved over an extended period under vacuum as long as the temperature remained well-below *T*_0_=170 K. The appreciable quantity of hydrogen stored at this low pressure and low temperature condition was then measured by returning the system to ambient temperature (Fig. 6a) thereby completely releasing the stored H_2_. The cumulative amount of H_2_ evolved varied with the initial dosing pressure, which for 10 MPa amounted to about 0.95 wt%. This result demonstrates the ability to store physically substantial quantities of H_2_ in materials with temperature-regulated pore accessibility without sustained external pressure. The encapsulation capacity is proportional to the intracrystal pore volume as opposed to the surface area: in this example the H_2_ encapsulated was 30 times greater than the maximum adsorption capacity at the same storage temperature. These preliminary results suggest the possibility of storing 3–7 wt% H_2_ at ambient pressures by quenching a chabazite initially dosed at ambient temperature to 70–250 MPa (Supplementary Note 6 and Supplementary Fig. 21). A potential deployment method for the on-board fuel gas storage via gas encapsulation has been exemplified in Supplementary Fig. 22. The encapsulation temperature could be elevated to near ambient by using heavier door-keeping cations, for example, Rb^+^, while the storage capacity could be increased by designing a composite material with a high porosity core and a trapdoor shell. Figure 6b also shows the controlled release of H_2_ through the manipulation of system temperature, thereby enabling a nano-regulator for molecular transport. The encapsulation/decapsulation of CH_4_ was also demonstrated with trapdoor **r2**KCHA and supramolecular *p*-*t*-butylcalix[4]arene and visualized by igniting the CH_4_ being evolved from the materials, generating a sustained or abrupt flame, respectively (Supplementary Movies 1 and 2).

## Discussion

We have elucidated a mechanism for the important but poorly understood phenomenon of temperature-regulated guest admission in several typical microporous materials. Such admission processes involve the temporary and reversible displacement of a pore-blocking group as a result of interactions with the guest molecule. The adsorption model developed here to describe the dependence of guest admission on temperature for four gases on three classes of materials allows for robust quantification of this effect, which has not been possible to date. The variation in the admission threshold temperature with the guest's size offers new opportunities for thermo-regulated sieving applications. Similarly, the dependence in the width of the pore accessibility distribution on the guest's molecular mass has the potential to enable sophisticated gas sensing and separation processes. With the trapdoor zeolite, we also demonstrated the ability to physically encapsulate and store appreciable amounts of H_2_ and CH_4_ without sustained external pressure, and the ability to regulate its release by thermally controlled decapsulation. These findings should help illuminate new paths for gas and energy storage by molecular encapsulation.

## Methods

### Synthesis of microporous samples

Chabazite with Si/Al ratio of 2.2 was synthesized from zeolite Y following the reported method^16^, and then ion exchanged to the potassium form with a typical procedure: 5 g of chabazite in 200 ml of 1 M KCl was refluxed at 343 K under stirring for 24 h, followed by filtration and washing with deionized water 2–3 times. The above ion exchange procedure was repeated twice to obtain fully exchanged potassium chabazite (labelled as **r2**KCHA). *p*-*t*-butylcalix[4]arene was synthesized according to the reported procedure^27^,^28^. After the synthesis, the sample was heated under vacuum at 140 °C for 24 h to completely remove any possible entrapped solvent toluene from the calixarene cavity, ensuring it was vacant. The sample was sublimed at 280 °C under vacuum to give the final product used in the analysis^49^.

### Sample characterization

The species of inorganic ions and Si/Al ratios of the chabazites were determined by inductively coupled plasma-mass spectroscopy. For *in situ* synchrotron PXRD experiments, the zeolite powder was degassed on Micromeritics ASAP 2010 (623 K for 12 h below 1 Pa), transferred to a 0.7 mm quartz capillary, and sealed in an Ar glove bag. Then the capillary was decanted at one end and mounted on the synchrotron PXRD machine with the open end being connected to vacuum/gas line. Before the experiments, the sample was re-degassed *in situ*, followed by cooling to 213 K. For the scans under vacuum, the sample was heated from 213 to 363 K at 5 K min^−1^, during which XRD data were collected at a series of temperature points; the sample was held at each set point for 60 s before scanning for 600 s. For the scans in N_2_, a similar procedure was adopted and the only difference is that N_2_ was dosed into the capillary slowly to 1 bar at 423 K before cooling to 213 K and the N_2_ pressure was kept at 1 bar. N_2_ was introduced at a relatively high temperature to assure the gas was admitted and the behaviour of the zeolite sample truly reflected the presence of the gas. A Mythen-II detector was used for data collection with a X-ray wavelength of 0.5894 Å for all zeolites.

The purity of *p*-*t*-butylcalix[4]arene was verified by nuclear magnetic resonance (Supplementary Figs 23 and 24) and high performance liquid chromatography, and no impurities were detected by either technique. The crystal structure of the *p*-*t*-butylcalix[4]arene was also identified by synchrotron PXRD with an X-ray wavelength of 0.77502 Å (Supplementary Fig. 25).

### Gas adsorption measurements

Before the isotherm measurements, the samples were thoroughly dehydrated and degassed by heating stepwise to 413 K for *p*-*t*-butylcalix[4]arene and 623 K for **r2**KCHA under high vacuum overnight on a Micromeritics ASAP2010/2020 analyser. After degas the samples were cooled to room temperature and backfilled with He. Adsorption isotherms on **r2**KCHA were measured in the temperature range of 77–343 K for N_2_, 77–303 K for H_2_, 197–305 K for Ar and 195–353 K for CH_4_ and at pressures up to 120 kPa. Temperatures of 77 and 195 K were achieved with liquid nitrogen and ethanol-dry ice baths, respectively. Other non-standard cryogenic temperatures below 243 K were achieved using a solid–liquid thermostatic bath of water–ethanol in different ratios. CH_4_ isotherms on *p*-*t*-butylcalix[4]arene were measured in the temperature range from 195 to 323 K. The rate of adsorption of CH_4_ on **r2**KCHA was measured at 333, 279 and 195 K using the reported procedure and equipment^50^, representing the fully pore accessible, transitional and pore inaccessible temperature ranges, respectively.

### Density functional theory calculations

*Ab initio* DFT calculations were employed to determine the energy barriers associated with the guest admission process as well as the snapshots of the trajectories of the gas molecules and the door-keeping cations in **r2**KCHA. The Vienna *Ab initio* Simulation Package^51^ was used with the projector augmented waves approach^52^ and the dispersion-corrected DFT-D3 functional^53^.

### Grand Canonical Monte Carlo calculations

Adsorption isotherms for CH_4_ and N_2_ at all temperatures were computed by the GCMC method using the RASPA software^35^,^36^. The structures of the trapdoor **r2**KCHA determined from synchrotron PXRD experiments were used to construct the molecular model for GCMC. A 3 × 3 × 3 supercell was built to calculate adsorption equilibria for blocked pores. See Supplementary Note 4 for simulation details, and the Crystallographic Information Files of a cubic unit cell and the supercell appended separately.

### Hydrogen and methane encapsulation

The experimental procedure illustrated in Fig. 6a used 5.93 g of **r2**KCHA with dosing and quenching temperatures of 323 and 77 K, respectively. The setup for conducting the reversible hydrogen encapsulation and decapsulation experiment is detailed in Supplementary Fig. 20. No activation or degas was needed between repeated cycles. In a similar setup, the encapsulation/decapsulation of methane with both **r2**KCHA (6.06 g) and *p*-*t*-butylcalix[4]arene (3.27 g) was demonstrated by igniting the CH_4_ evolved (Supplementary Movies 1 and 2).

### Data availability

All data generated or analysed during this study are included in this published article (and its Supplementary Information Files). The Crystallographic Information File data are available in the Dryad Digital Repository http://datadryad.org/ via doi:10.5061/dryad.2426m.

## Additional information

**How to cite this article:** Li, G. *et al*. Temperature-regulated guest admission and release in microporous materials. *Nat. Commun.* **8,** 15777 doi: 10.1038/ncomms15777 (2017).

**Publisher's note:** Springer Nature remains neutral with regard to jurisdictional claims in published maps and institutional affiliations.

## Supplementary Material

Supplementary Information Supplementary Figures, Supplementary Tables, Supplementary Notes and Supplementary References

Supplementary Movie 1 Ignition of methane evolved from trapdoor chabazites generating a sustained flame.

Supplementary Movie 2 Ignition of methane evolved from p-t-butylcalix[4]arene generating an abrupt flame.

Peer Review File

## References

[b1] Herm Z. R. . Separation of hexane isomers in a metal-organic framework with triangular channels. Science 340, 960–964 (2013). 23704568 10.1126/science.1234071

[b2] Kuznicki S. M. . A titanosilicate molecular sieve with adjustable pores for size-selective adsorption of molecules. Nature 412, 720–724 (2001). 11507636 10.1038/35089052

[b3] Moliner M., Martínez C. & Corma A. Synthesis strategies for preparing useful small pore zeolites and zeotypes for gas separations and catalysis. Chem. Mater. 26, 246–258 (2013).

[b4] Hudson M. R. . Unconventional, highly selective CO_2_ adsorption in zeolite SSZ-13. J. Am. Chem. Soc. 134, 1970–1973 (2012). 22235866 10.1021/ja210580b

[b5] Nugent P. . Porous materials with optimal adsorption thermodynamics and kinetics for CO_2_ separation. Nature 495, 80–84 (2013). 23446349 10.1038/nature11893

[b6] Murray L. J., Dinca M. & Long J. R. Hydrogen storage in metal-organic frameworks. Chem. Soc. Rev. 38, 1294–1314 (2009). 19384439 10.1039/b802256a

[b7] Kuc A., Zhechkov L., Patchkovskii S., Seifert G. & Heine T. Hydrogen sieving and storage in fullerene intercalated graphite. Nano Lett. 7, 1–5 (2007). 17212430 10.1021/nl0619148

[b8] Davis M. E. Ordered porous materials for emerging applications. Nature 417, 813–821 (2002). 12075343 10.1038/nature00785

[b9] Bein T., Brown K., Frye G. C. & Brinker C. J. Molecular sieve sensors for selective detection at the nanogram level. J. Am. Chem. Soc. 111, 7640–7641 (1989).

[b10] Auerbach S. M., Carrado K. A. & Dutta P. K. Handbook of zeolite science and technology CRC (2003).

[b11] Nair S., Tsapatsis M., Toby B. H. & Kuznicki S. M. A study of heat-treatment induced framework contraction in strontium-ETS-4 by powder neutron diffraction and vibrational spectroscopy. J. Am. Chem. Soc. 123, 12781–12790 (2001). 11749535 10.1021/ja011703z

[b12] Li J.-R., Kuppler R. J. & Zhou H.-C. Selective gas adsorption and separation in metal-organic frameworks. Chem. Soc. Rev. 38, 1477–1504 (2009). 19384449 10.1039/b802426j

[b13] Zhao D., Yuan D., Krishna R., van Baten J. M. & Zhou H.-C. Thermosensitive gating effect and selective gas adsorption in a porous coordination nanocage. Chem. Commun. 46, 7352–7354 (2010). 10.1039/c0cc02771e 20820545

[b14] Lyndon R. . Dynamic photo-switching in metal-organic frameworks as a route to low-energy carbon dioxide capture and release. Angew. Chem. Int. Ed. 52, 3695–3698 (2013). 10.1002/anie.201206359 23401101

[b15] Breck D. W., Eversole W. G., Milton R. M., Reed T. B. & Thomas T. L. Crystalline zeolites. I. the properties of a new synthetic zeolite, type A. J. Am. Chem. Soc. 78, 5963–5972 (1956).

[b16] Shang J. . Discriminative separation of gases by a ‘molecular trapdoor' mechanism in chabazite zeolites. J. Am. Chem. Soc. 134, 19246–19253 (2012). 23110556 10.1021/ja309274y

[b17] De Baerdemaeker T. & De Vos D. Gas separation: trapdoors in zeolites. Nat. Chem. 5, 89–90 (2013). 23344444 10.1038/nchem.1560

[b18] Ma S., Sun D., Wang X.-S. & Zhou H.-C. A mesh-adjustable molecular sieve for general use in gas separation. Angew. Chem. Int. Ed. 46, 2458–2462 (2007). 10.1002/anie.200604353 17318932

[b19] Ma S., Sun D., Yuan D., Wang X.-S. & Zhou H.-C. Preparation and gas adsorption studies of three mesh-adjustable molecular sieves with a common structure. J. Am. Chem. Soc. 131, 6445–6451 (2009). 19379001 10.1021/ja808896f

[b20] Kim H. . Temperature-triggered gate opening for gas adsorption in microporous manganese formate. Chem. Commun. 4697–4699 (2008). 10.1039/b811087e 18830463

[b21] Deng H., Olson M. A., Stoddart J. F. & Yaghi O. M. Robust dynamics. Nat. Chem. 2, 439–443 (2010). 20489710 10.1038/nchem.654

[b22] Gao Q., Xu J., Cao D., Chang Z. & Bu X. H. A rigid nested metal-organic framework featuring a thermoresponsive gating effect dominated by counterions. Angew. Chem. Int. ed. 55, 15027–15030 (2016). 10.1002/anie.201608250 27791310

[b23] Shang J. . Determination of composition range for ‘molecular trapdoor' effect in chabazite zeolite. J. Phys. Chem. C 117, 12841–12847 (2013).

[b24] Lozinska M. M. . Understanding carbon dioxide adsorption on univalent cation forms of the flexible zeolite Rho at conditions relevant to carbon capture from flue gases. J. Am. Chem. Soc. 134, 17628–17642 (2012). 23013547 10.1021/ja3070864

[b25] Lozinska M. M. . Cation gating and relocation during the highly selective ‘trapdoor' adsorption of CO_2_ on univalent cation forms of zeolite Rho. Chem. Mater. 26, 2052–2061 (2014).

[b26] Remy T. . Adsorption and separation of CO_2_ on KFI zeolites: effect of cation type and Si/Al ratio on equilibrium and kinetic properties. Langmuir 29, 4998–5012 (2013). 23509898 10.1021/la400352r

[b27] Thallapally P. K., Kirby K. A. & Atwood J. L. Comparison of porous and nonporous materials for methane storage. New J. Chem. 31, 628–630 (2007).

[b28] Atwood J. L., Barbour L. J., Thallapally P. K. & Wirsig T. B. A crystalline organic substrate absorbs methane under STP conditions. Chem. Commun. 51–53 (2005). 10.1039/b416752j 15614369

[b29] Yang R. T. Gas separation by adsorption processes Vol. 3 (Imperial College Press (1997).

[b30] Do D. D. Adsorption analysis: equilibria and kinetics Ch. 5 (Imperial College Press (1998).

[b31] Breck D. W. Zeolite molecular sieves: structure, chemistry, and use Wiley (1974).

[b32] Breck D. W. Crystalline molecular sieves. J. Chem. Edu. 41, 678 (1964).

[b33] Miller W., Smith C. W., Mackenzie D. S. & Evans K. E. Negative thermal expansion: a review. J. Mater. Sci. 44, 5441–5451 (2009).

[b34] Queen W. L. . Site-specific CO_2_ adsorption and zero thermal expansion in an anisotropic pore network. J. Phys. Chem. C 115, 24915–24919 (2011).

[b35] Dubbeldam D., Calero S., Ellis D. E. & Snurr R. Q. RASPA: molecular simulation software for adsorption and diffusion in flexible nanoporous materials. Mol. Simul. 42, 81–101 (2016).

[b36] Dubbeldam D., Torres-Knoop A. & Walton K. S. On the inner workings of Monte Carlo codes. Mol. Simul. 39, 1253–1292 (2013).

[b37] Fang H. . Identification of high-CO_2_-capacity cationic zeolites by accurate computational screening. Chem. Mater. 28, 3887–3896 (2016).

[b38] Fang H. . First principles derived, transferable force fields for CO_2_ adsorption in Na-exchanged cationic zeolites. Phys. Chem. Chem. Phys. 15, 12882–12894 (2013). 23807115 10.1039/c3cp52246f

[b39] Vékey K. Internal energy effects in mass spectrometry. J. Mass Spectrom. 31, 445–463 (1996).

[b40] Drahos L. & Vékey K. Determination of the thermal energy and its distribution in peptides. J. Am. Soc. Mass Spectrom. 10, 323–328 (1999).

[b41] Myers A. L. & Prausnitz J. M. Thermodynamics of mixed-gas adsorption. AIChE J. 11, 121–127 (1965).

[b42] Kapoor A., Ritter J. A. & Yang R. T. An extended Langmuir model for adsorption of gas-mixtures on heterogeneous surfaces. Langmuir 6, 660–664 (1990).

[b43] Li G., Xiao P. & Webley P. Binary adsorption equilibrium of carbon dioxide and water vapor on activated alumina. Langmuir 25, 10666–10675 (2009). 19678623 10.1021/la901107s

[b44] Physick A. J. W. . Novel low energy hydrogen-deuterium isotope breakthrough separation using a trapdoor zeolite. Chem. Eng. J. 288, 161–168 (2016).

[b45] Ananchenko G. S. . A molecular turnstile in para-octanoyl calix[4]arene nanocapsules. Chem. Commun. 707–709 (2007). 10.1039/b613972h 17392957

[b46] Zhang J.-P. & Chen X.-M. Exceptional framework flexibility and sorption behavior of a multifunctional porous cuprous triazolate framework. J. Am. Chem. Soc. 130, 6010–6017 (2008). 18386891 10.1021/ja800550a

[b47] Liu B. & Smit B. Comparative molecular simulation study of CO_2_/N_2_ and CH_4_/N_2_ separation in zeolites and metal-organic frameworks. Langmuir 25, 5918–5926 (2009). 19382791 10.1021/la900823d

[b48] Li G. . Hydrogen storage in Pd nanocrystals covered with a metal-organic framework. Nat. Mater. 13, 802–806 (2014). 25017188 10.1038/nmat4030

[b49] Atwood J. L., Barbour L. J., Jerga A. & Schottel B. L. Guest transport in a nonporous organic solid via dynamic van der Waals cooperativity. Science 298, 1000–1002 (2002). 12411698 10.1126/science.1077591

[b50] Xiao G., Li Z., Saleman T. L. & May E. F. Adsorption equilibria and kinetics of CH_4_ and N_2_ on commercial zeolites and carbons. Adsorption 23, 131–147 (2016).

[b51] Kresse G. & Furthmüller J. Efficient iterative schemes for ab initio total-energy calculations using a plane-wave basis set. Phys. Rev. B 54, 11169–11186 (1996). 10.1103/physrevb.54.11169 9984901

[b52] Kresse G. & Joubert D. From ultrasoft pseudopotentials to the projector augmented-wave method. Phys. Rev. B 59, 1758–1775 (1999).

[b53] Grimme S. . A consistent and accurate *ab initio* parametrization of density functional dispersion correction (DFT-D) for the 94 elements H-Pu. The Journal of Chemical Physics. 132, 154104 (2010). 20423165 10.1063/1.3382344

