# Supplementary material for: Temperature-regulated guest admission and release in microporous materials
Source: Nat Commun. 2017 Jun 9;8:15777. doi: 10.1038/ncomms15777 (PMC5472718; doi:10.1038/ncomms15777)
Supplement: Supplementary Information — Supplementary Figures, Supplementary Tables, Supplementary Notes and Supplementary References [file ncomms15777-s1.pdf]

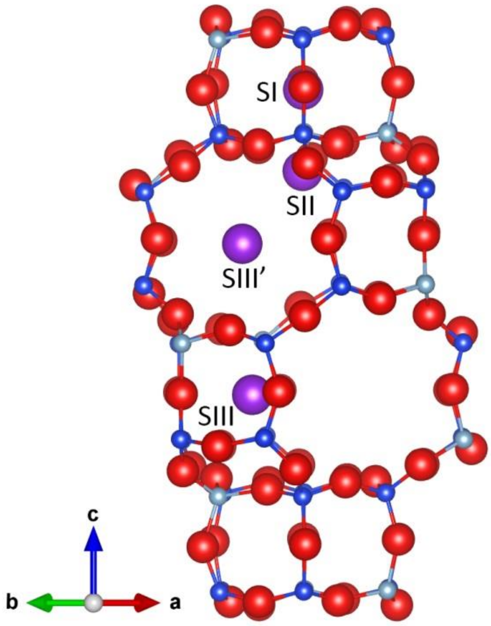

Supplementary Figure 1 | Chabazite structure and cation sites. Schematic representation of a chabazite unit cell structure and the occupancy sites for extra-framework cations. Double-six ring prisms (D6Rs) connected by tilted four-membered rings (4MRs) form a three-dimensional structure, creating eight membered rings (8MRs) as the only access to the crystal interior. Four types of cation sites exist: at the centre of a D6R (SI); above a D6R inside the cavity (SII); next to a 4MR of a D6R inside the cavity (SIII); at the centre of an 8MR (SIII'). Large cations that preferentially locate at SIII's are pore-keeping cations. Colour code: K in violet, Al in dark grey, Si in blue, and O in red.

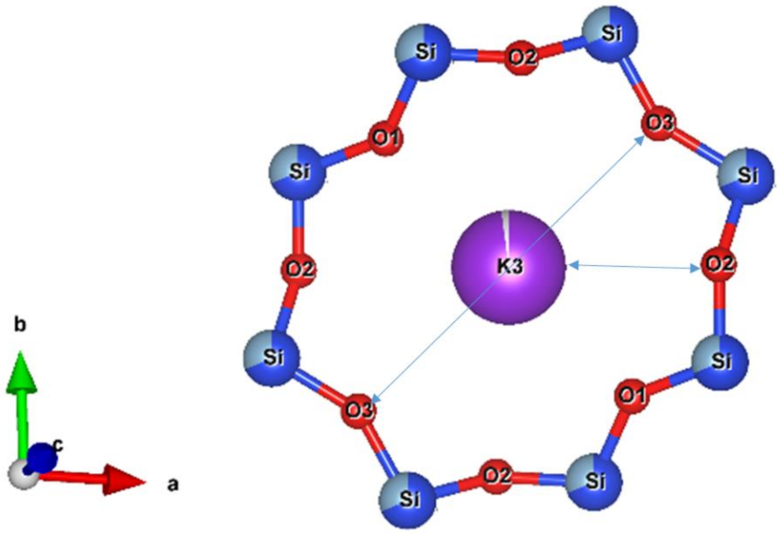

Supplementary Figure 2 | Illustration of the 8MR pore framework and the pore-keeping cation at site SIII'. The dimension of the 8MR pore is characterized in three directions, namely D_1 for the distance between (O1-O1), D_2 for the distance between (O2-O2), and D_3 for (O3-O3). Atomic size not in portion. The exact locations of each atoms and cation were determined by Rietveld refinement of the synchrotron PXRD data.

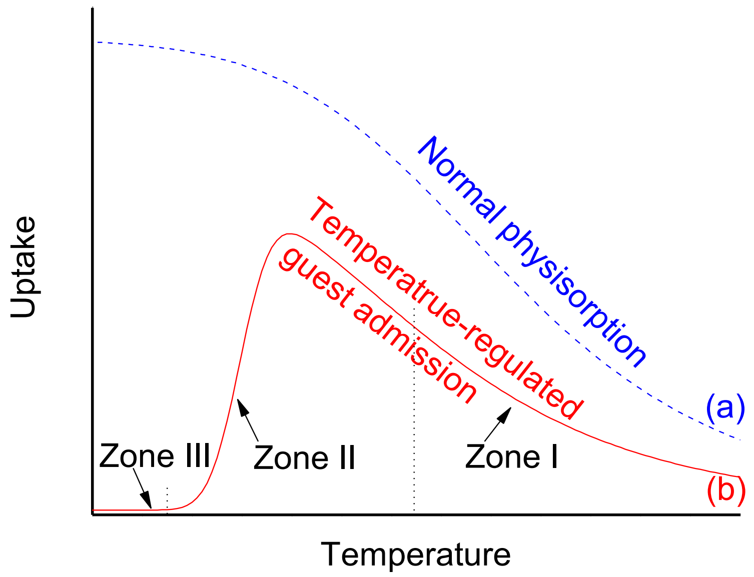

Supplementary Figure 3 | Comparison of isobars for normal physisorption and temperature-dependent admission. Illustration of a “normal” *physisorption* isobar (blue dashed line) showing a monotonic increase in uptake with decreasing temperature (a), in comparison with an “abnormal” bell-shaped isobar for temperature-dependent guest admission observed on typical molecular trapdoor zeolites, showing an abrupt drop of loading below a certain temperature (b). The bell-shaped isobar can be divided into three zones with decreasing temperature, namely pore-accessible, transitional, and pore-inaccessible, denoted as Zone I, II, and III, respectively.

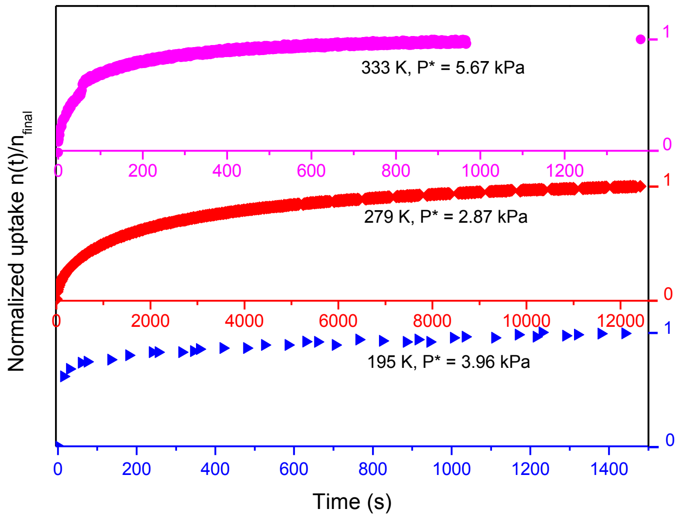

Supplementary Figure 4 | Uptake curves of CH_4 on r2KCHA at temperatures characteristic of the three different accessibility regimes.

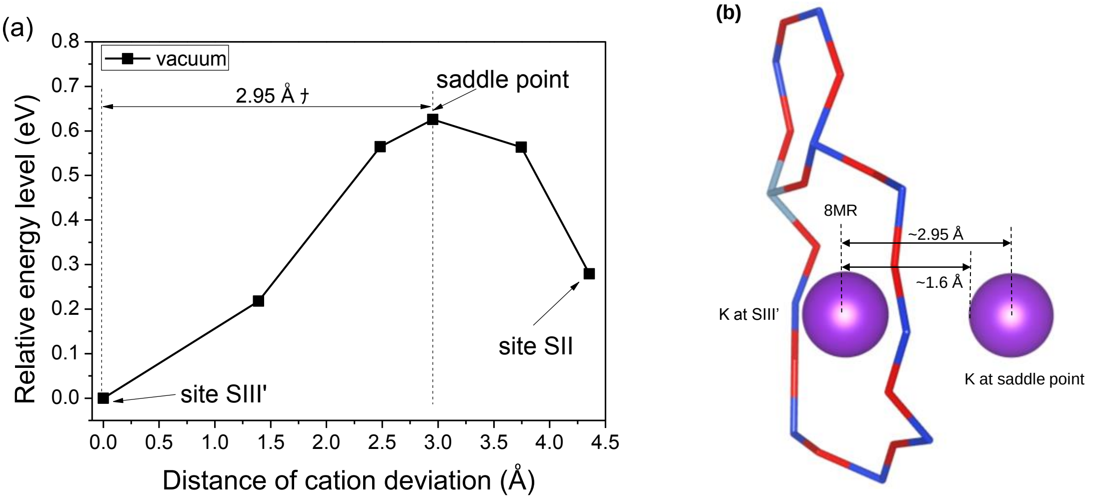

Supplementary Figure 5 | The energy profile for cation migration following SIII'-to-SII path. The distance from K cation at saddle point to K cation at site SIII' is ~2.95 Å. As the K cation at saddle point is along the 8MR passage, leaving a "room" of ~1.6 Å for guest molecule which is substantially smaller than the size of any gas molecule studied herein, the K cation has to pass the saddle point to allow for the entrance of the guest molecule.

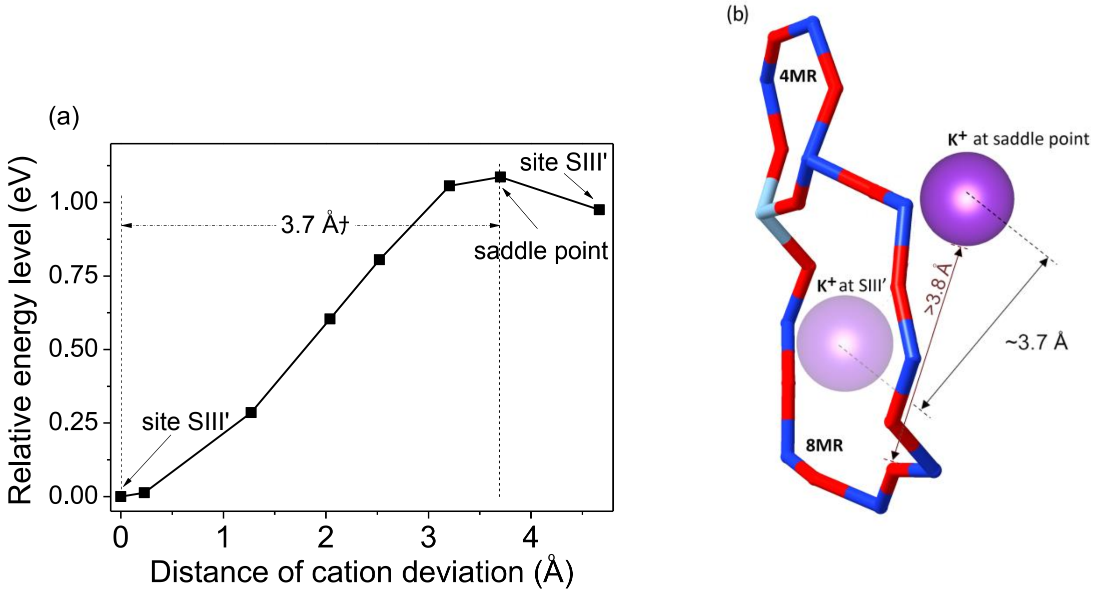

Supplementary Figure 6 | The energy profile for cation migration following SIII'-to-SIII path. The distance from K cation at saddle point to K cation at site SIII' is ~3.7 Å. As the position at saddle point is well beyond the 8MR passage, it gives a completely unblocked doorway (distance between K cation at saddle point and the opposite oxygen of the 8MR is greater than 3.8 Å) thus permitting the entrance of the guest molecule without the need for K cation necessarily reaching the saddle point.

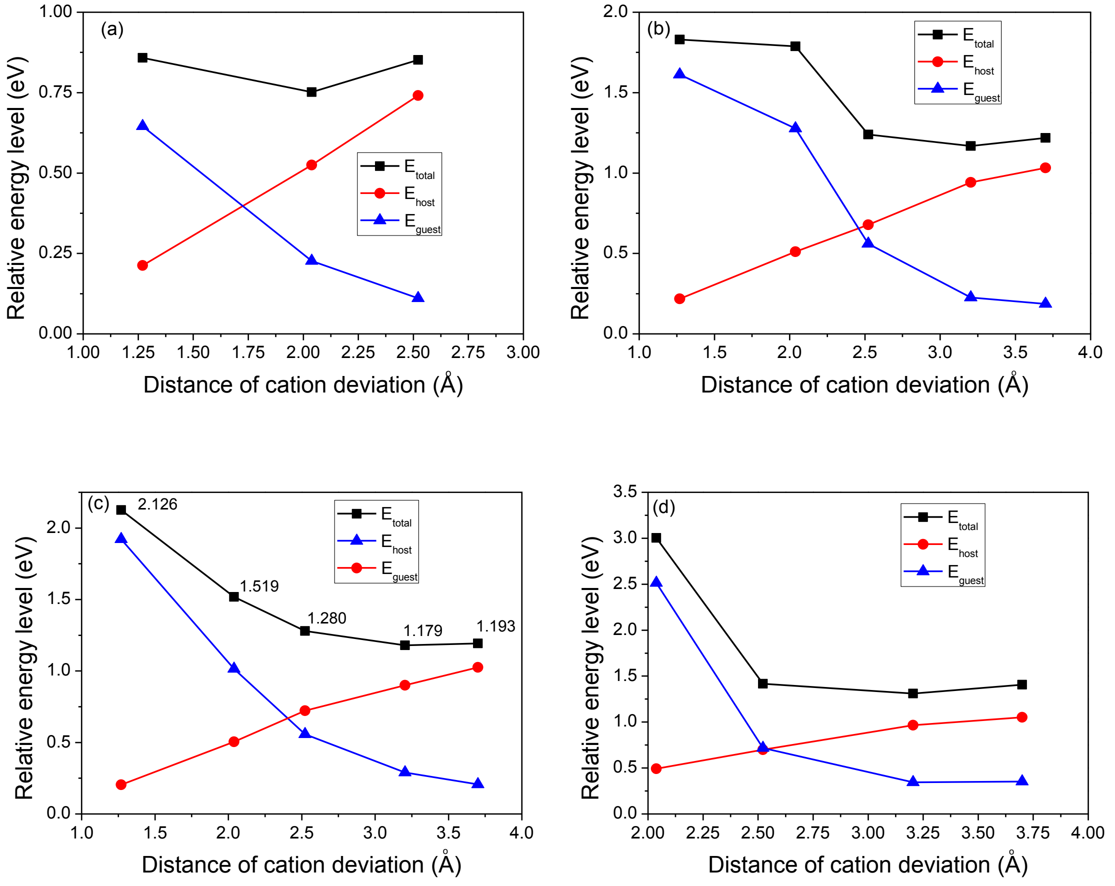

Supplementary Figure 7 | DFT-calculated energy barriers. Energy Barriers experienced by the door-keeping cation of the host zeolite, the interacting guest molecule during the gas admission into **r2**KCHA. (a) H_2, (b) Ar, (c) N_2, and (d) CH_4.

(a) 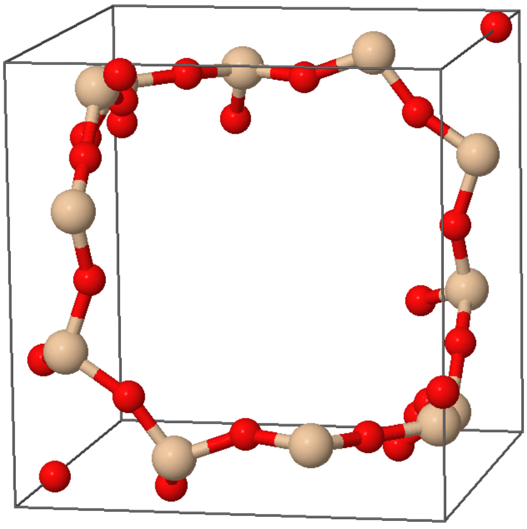 (b) 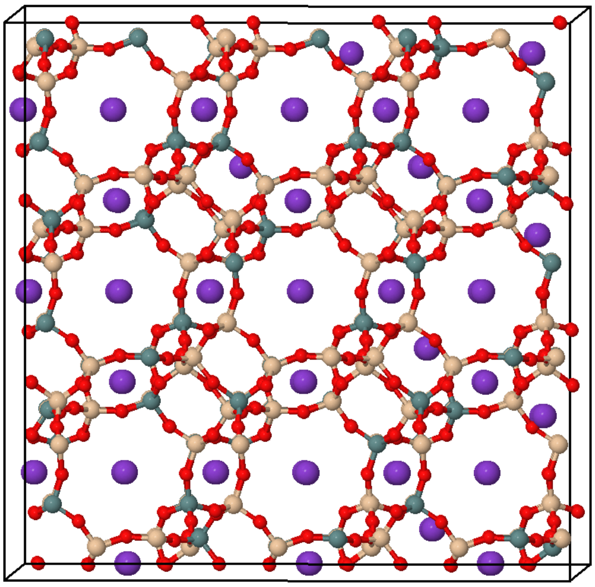

Supplementary Figure 8 | Unit cell and supercell model used for GCMC. (a) Experimental primitive unit cell of **r2**KCHA without K^+ ions. (b) 3 × 3 × 3 Supercell of experimental **r2**KCHA used in the GCMC calculations along with tempered K positions. Al, Si, O, and K atoms are depicted as dark teal, light brown, red, and violet spheres, respectively.

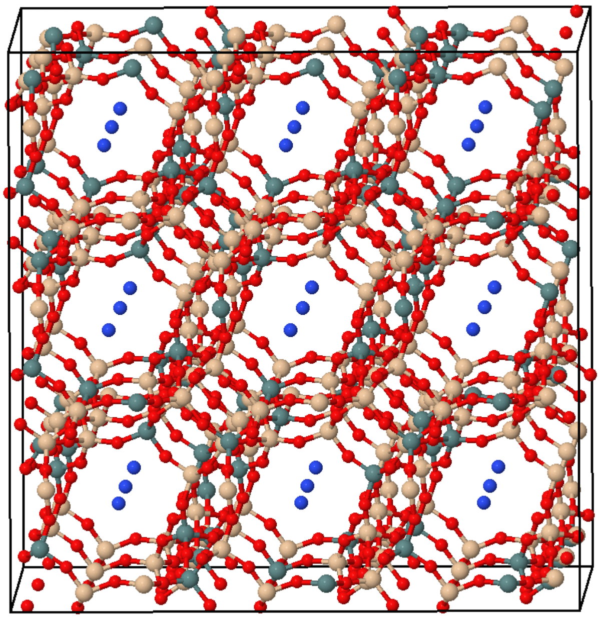

Supplementary Figure 9 | A 3 × 3 × 3 supercell of r2KCHA. The 27 cavity centres of **r2**KCHA are shown in the blue. Al, Si, and O atoms are depicted as dark teal, light brown, red, and violet spheres, respectively.

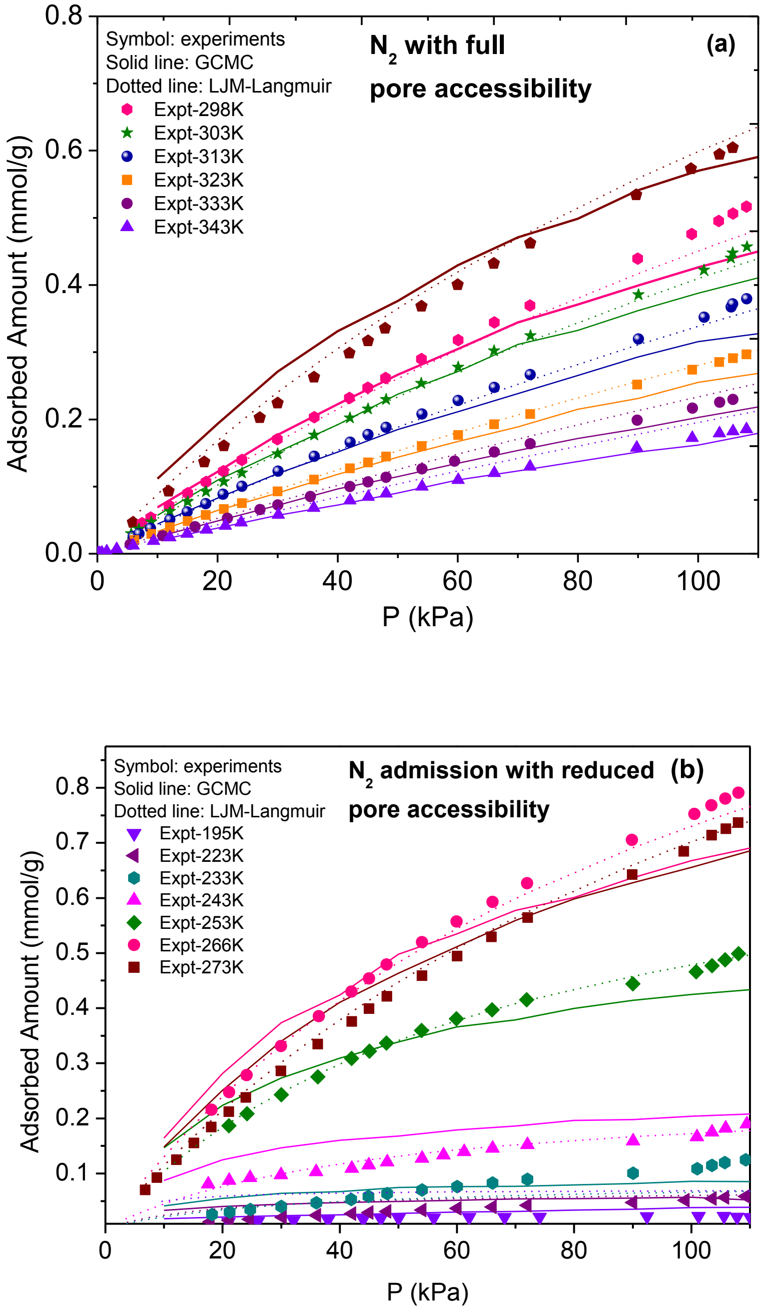

Supplementary Figure 10 | Comparison of simulated (using GCMC and LJM-Langmuir model) and experimental adsorption isotherms of N_2 on r2KCHA. In the temperature region with full pore accessibility (a), and reduced accessibility (b).

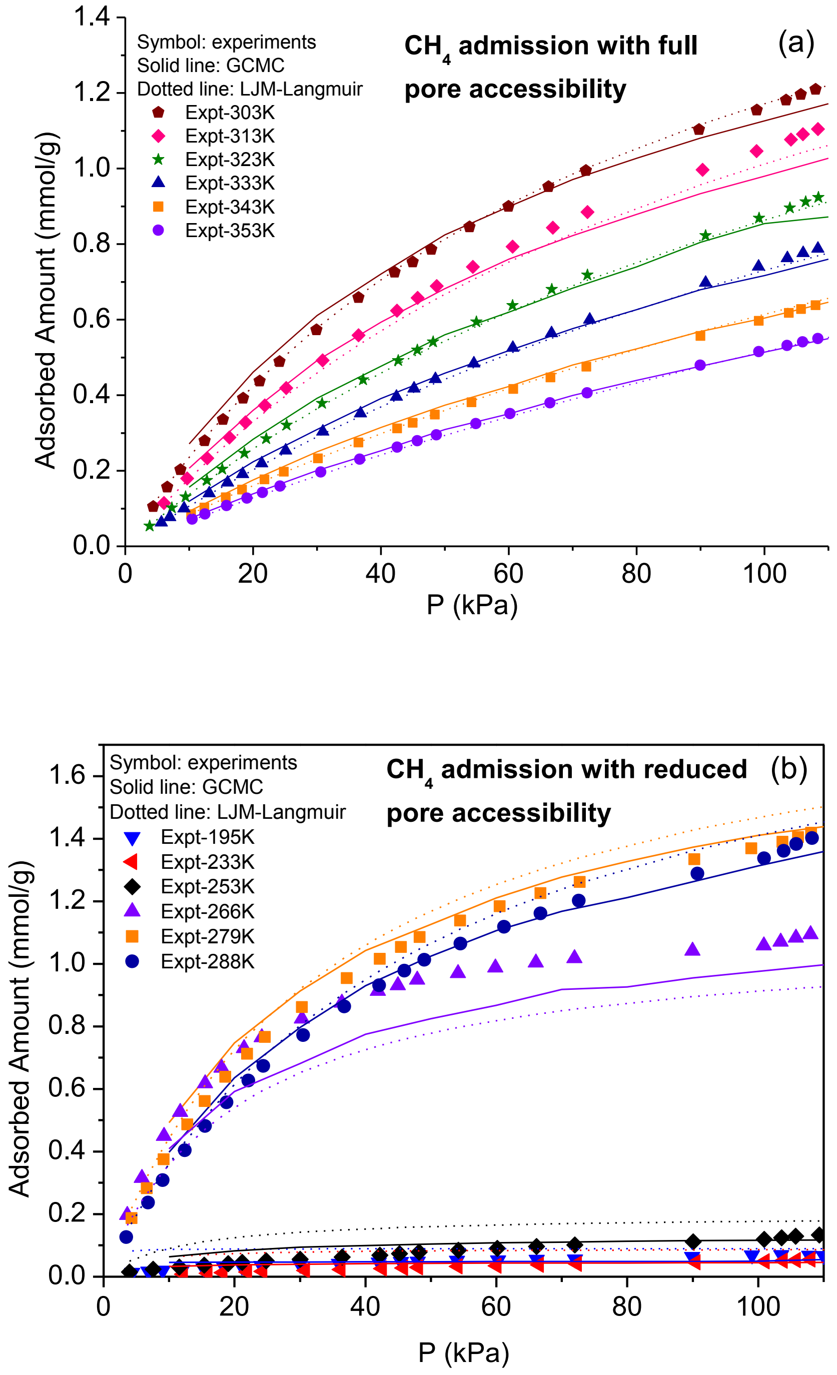

Supplementary Figure 11 | Comparison of simulated (using GCMC and LJM-Langmuir model) and experimental adsorption isotherms of CH_4 on r2KCHA. In the temperature region with full pore accessibility (a), and reduced accessibility (b).

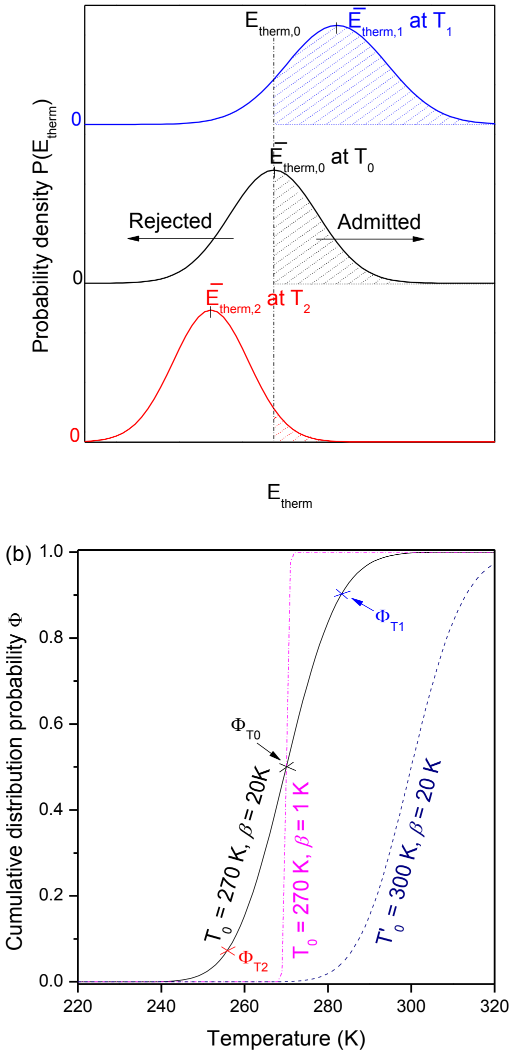

Supplementary Figure 12 | Illustration of Gaussian probability density function for thermal energies. At three different temperatures, with $T_1 > T_0 > T_2$, the higher the temperature, the wider the distribution. Shaded areas represent the portion of the system thermal energies that are greater than the threshold value $E_{\text{therm},0}$ (i.e. ΔE_{total})needed for guest admission. The mean thermal energy $\bar{E}_{\text{therm}}$ at temperature T_0 equals $E_{\text{therm},0}$. (b) Cumulative distribution function representing the fraction of accessible internal adsorption sites as calculated using eq.(5), where T_1, T_0, and T_2 were assigned as 283 K, 270 K, and 256 K, respectively. The black solid line corresponds to the standard distribution with T_0 = 270 K and β = 20 K; magenta dash-dotted line corresponds to the distribution with the same T_0 but a smaller β = 1 K, representing a step transition from non-accessible to accessible pores for the adsorbent unit cells; blue dashed line is for β = 20 K again but with a higher T_0 = 300 K showing the shift of pore-opening transition range to high temperatures.

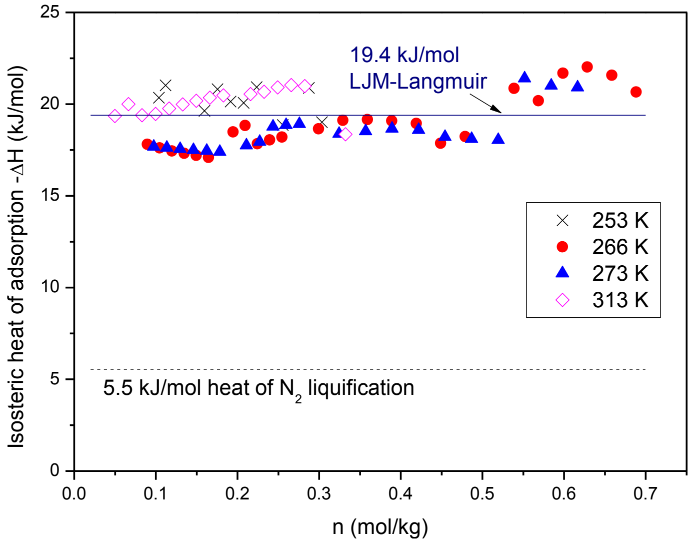

Supplementary Figure 13 | Isosteric heat of adsorption of N_2 on r2KCHA. Symbols are data points calculated from Clausius-Clapeyron equation by using saturation capacity determined from LJM-Langmuir at individual temperatures. Solid line denotes the isosteric heat (temperature independent) predicted by LJM-Langmuir model following thermodynamic van't Hoff equation.

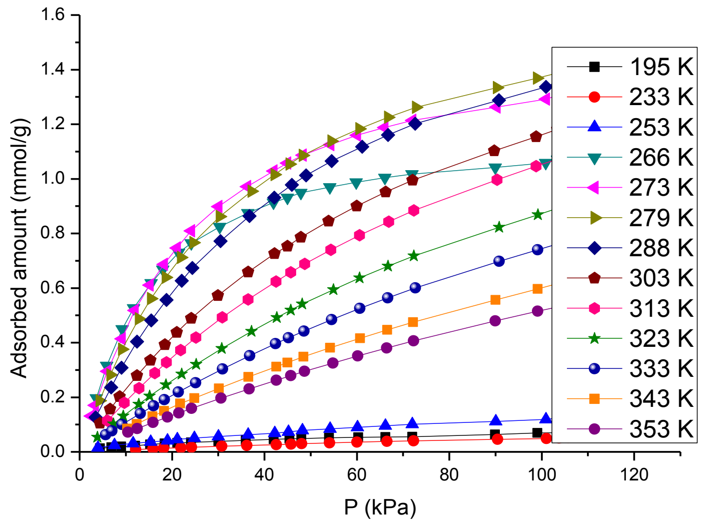

Supplementary Figure 14 | Adsorption isotherms of CH_4 on r2KCHA obtained experimentally in the temperature range of 195 – 353 K. The adsorption capacity first increases with decreasing temperature but then drops to nearly zero at low temperatures.

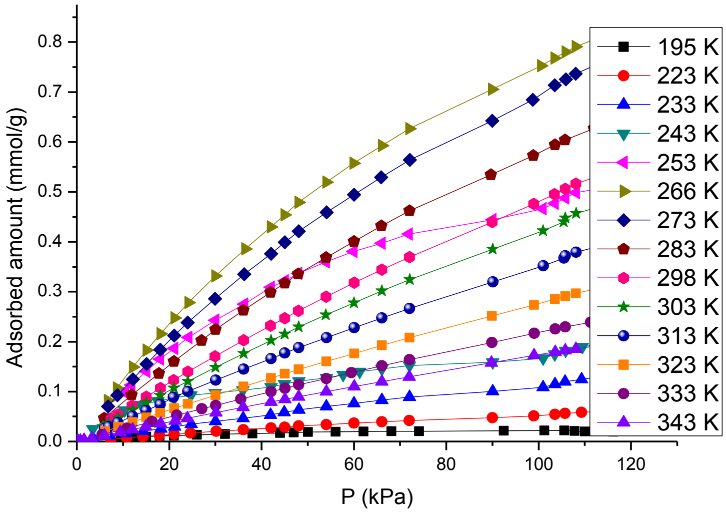

Supplementary Figure 15 | Adsorption isotherms of N_2 on r2KCHA obtained experimentally in the temperature range of 195 – 343 K. The adsorption capacity first increases with decreasing temperature but then drops to nearly zero at low temperatures.

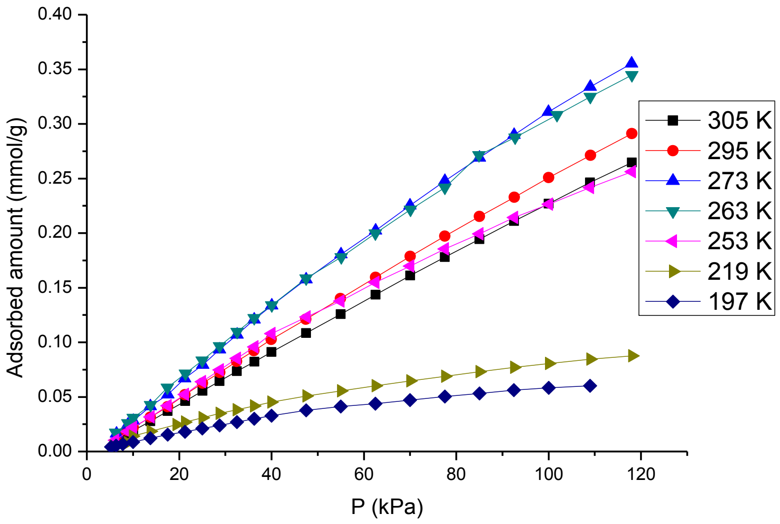

Supplementary Figure 16 | Adsorption isotherms of Ar on r2KCHA obtained experimentally in the temperature range of 197 – 305 K. The adsorption capacity first increases with decreasing temperature but then drops at lower temperatures.

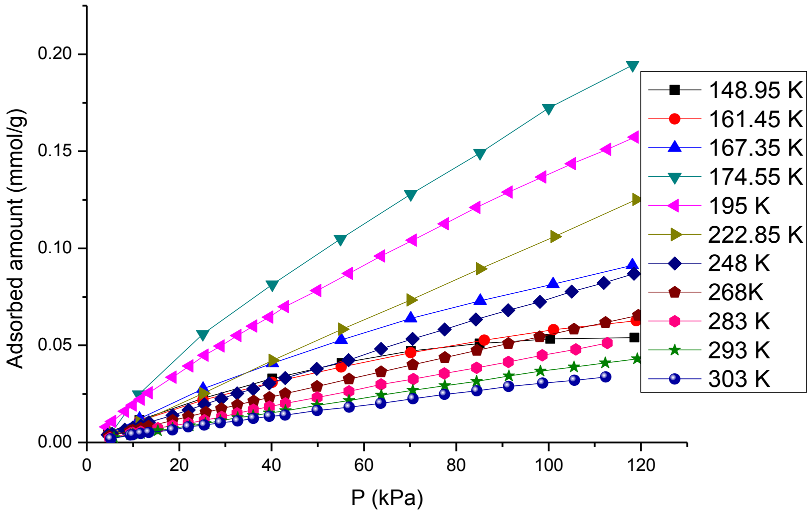

Supplementary Figure 17 | Adsorption isotherms of H_2 on r2KCHA obtained experimentally in the temperature range of 148.95 – 303 K. The adsorption capacity first increases with decreasing temperature but then drops at lower temperatures.

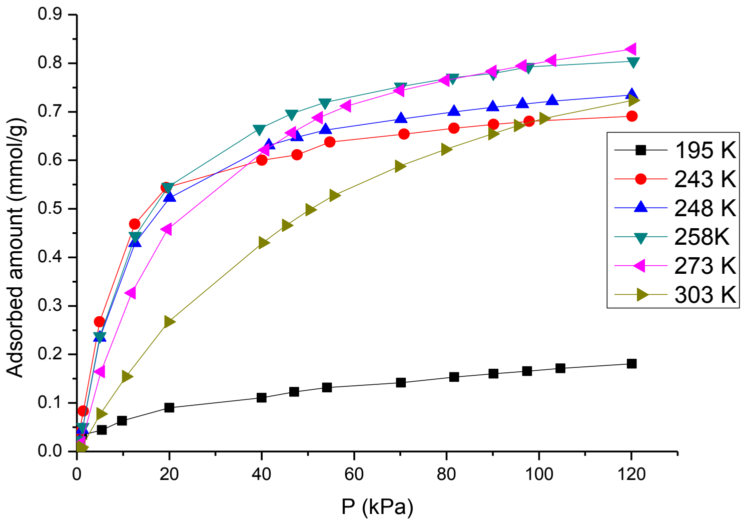

Supplementary Figure 18 | Adsorption isotherms of CH_4 on *p*-*t*-butylcalix[4]arene (CX[4]) obtained experimentally in the temperature range of 148.95 – 303 K. The adsorption capacity first increases with decreasing temperature but then drops at lower temperatures.

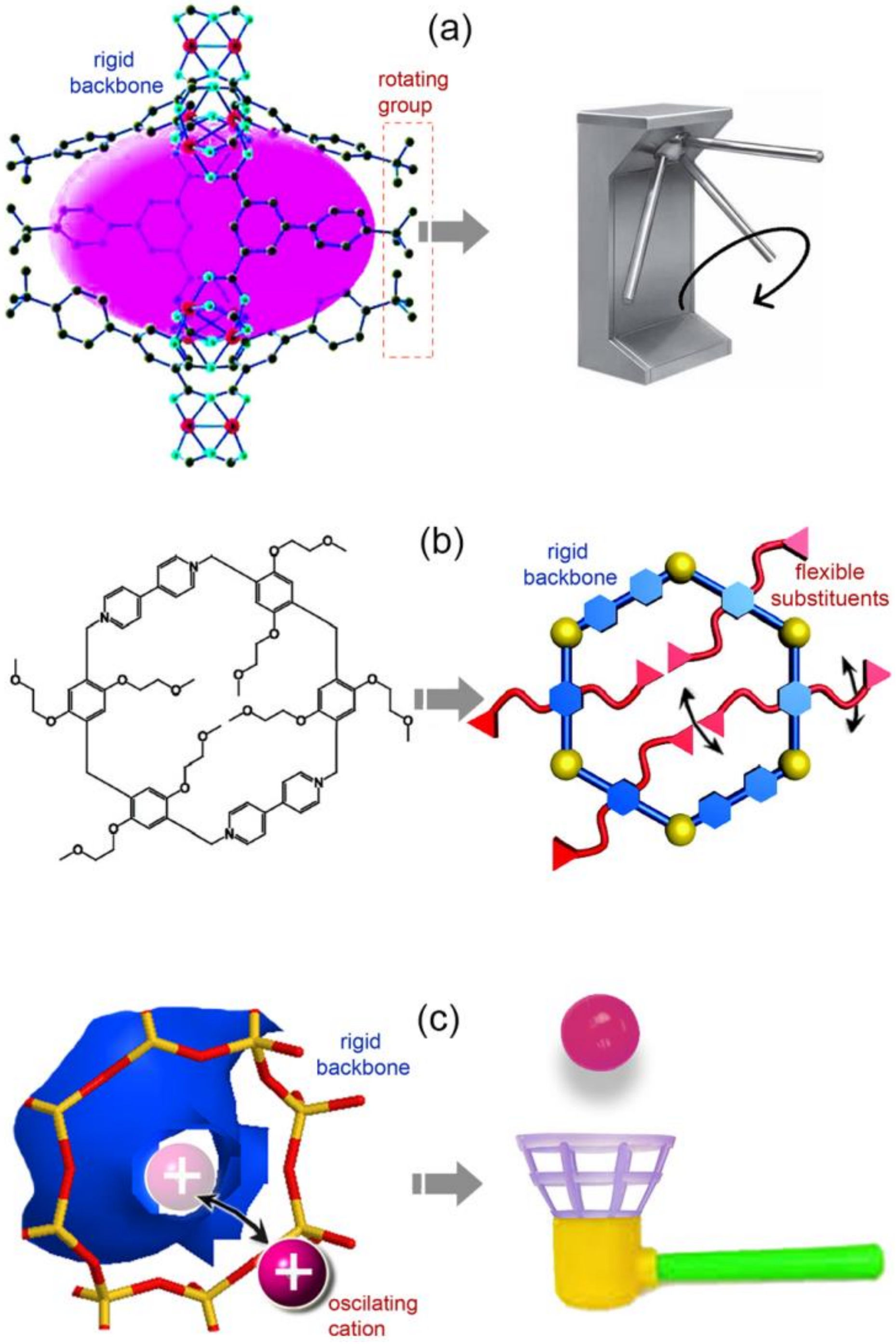

Supplementary Figure 19 | Notable examples of temperature-regulated guest admission. Schematic illustration of the structures of pore scaffolds and pore keeping groups of three typical microporous materials with temperature-regulated guest admission property, namely, (a) mesh-adjustable molecular sieve MOFs[1] with turnstile like pore-keeping groups, (b) a zinc-dicarboxylate-bipyridine MOF[2] with pores guarded by flexible side chains (Reproduced with permission from ref [2], Copyright 2010 American Chemical Society), and (c) an eight-membered ring (8MR) pore aperture of a molecular trapdoor zeolite showing the thermal oscillation of the pore keeping cation.

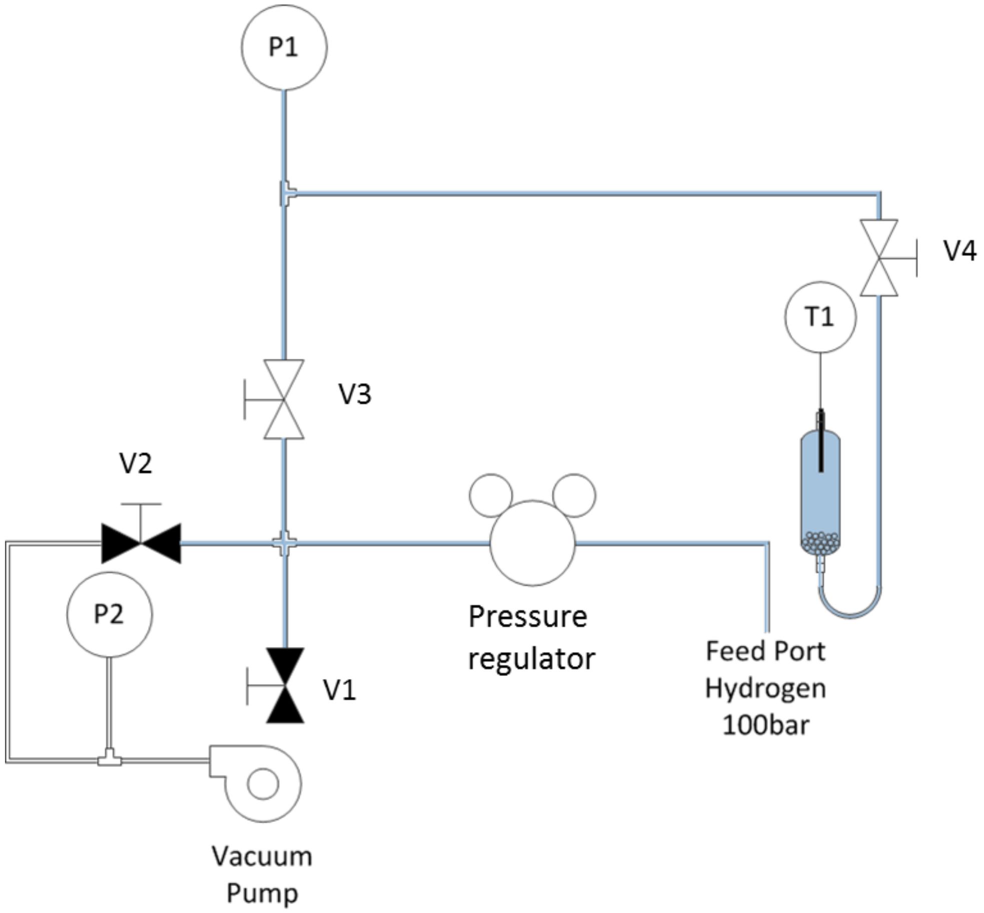

Supplementary Figure 20 | Schematic illustration of the setup of the encapsulation apparatus. The gas used for demonstration was hydrogen but can be other gases appropriate for the selected microporous material. The sample cylinder constructed for the purpose of containing the **r2**KCHA whilst it undergoes activation, dosing, quenching and decapsulation procedures. The main cylinder is a Swagelok double-ended TPED-compliant sample cylinder rated to 124 bar with 1/4 inch female NPT ends and a volume of 50cm^3. V-4 is a Swagelok stainless steel bonnet needle valve with 1/8 inch Swagelok tube fittings. Current valve positions show high pressure dosing step. At quenching step, the cylinder is immersed in a liquid nitrogen bath while keeping all valve positions unchanged and maintaining the gas pressure. At evacuation, the feed gas regulator is turned off and valve V1 is then opened for venting; after closing V1, valve V2 is opened to allow for evacuation of free space hydrogen from the dosed sample cylinder by the vacuum pump. The vacuum was maintained at a pressure below 1 mbar for approximately 10 min. The gas encapsulated inside the **r2**KCHA sample is then released by warming up the sample cylinder in ambient air; and the amount of gas evolved is quantified by measuring the pressure increase in the system.

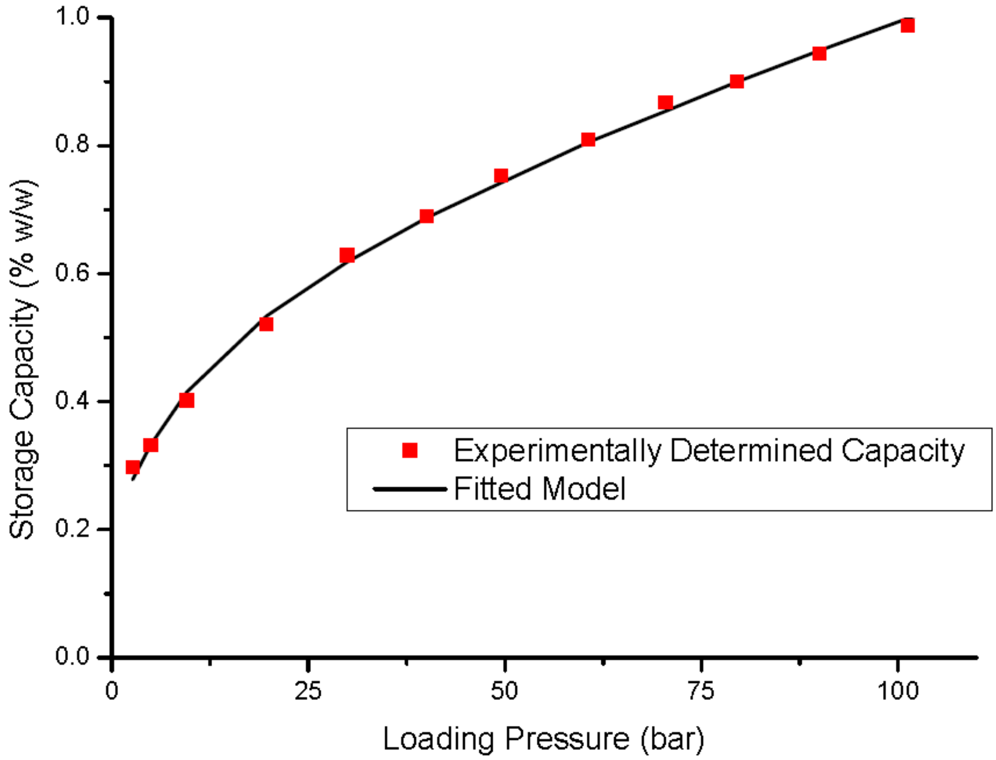

Supplementary Figure 21 | Hydrogen storage capacity on r2KCHA by molecular encapsulation as a function of initial dosing pressure.

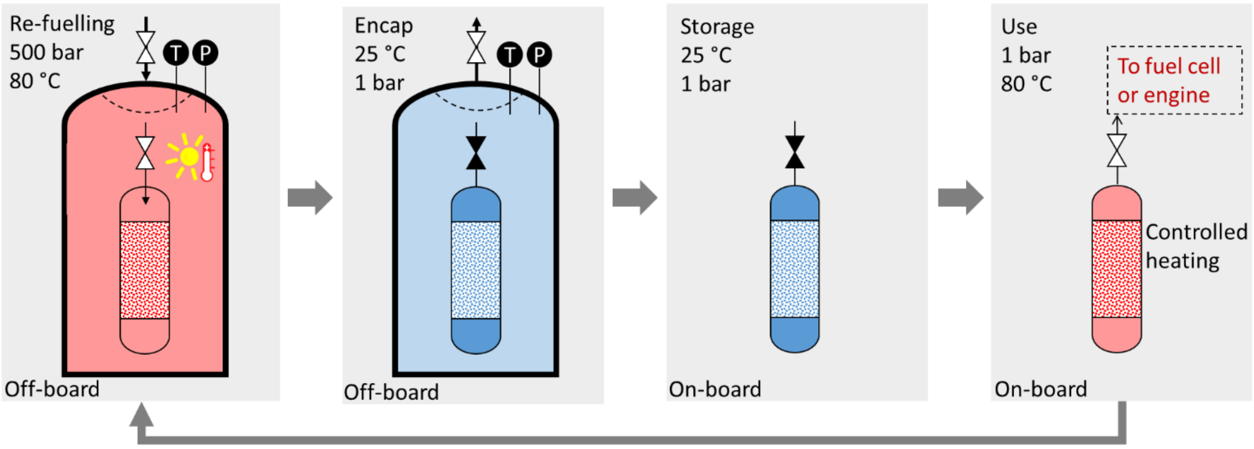

Supplementary Figure 22 | Proposed hydrogen filling, storage, use and re-fuelling mechanism for the implementation of on-board hydrogen storage via hydrogen encapsulation. The on-board tank is low pressure rated while the large dosing tank is high pressure rated.

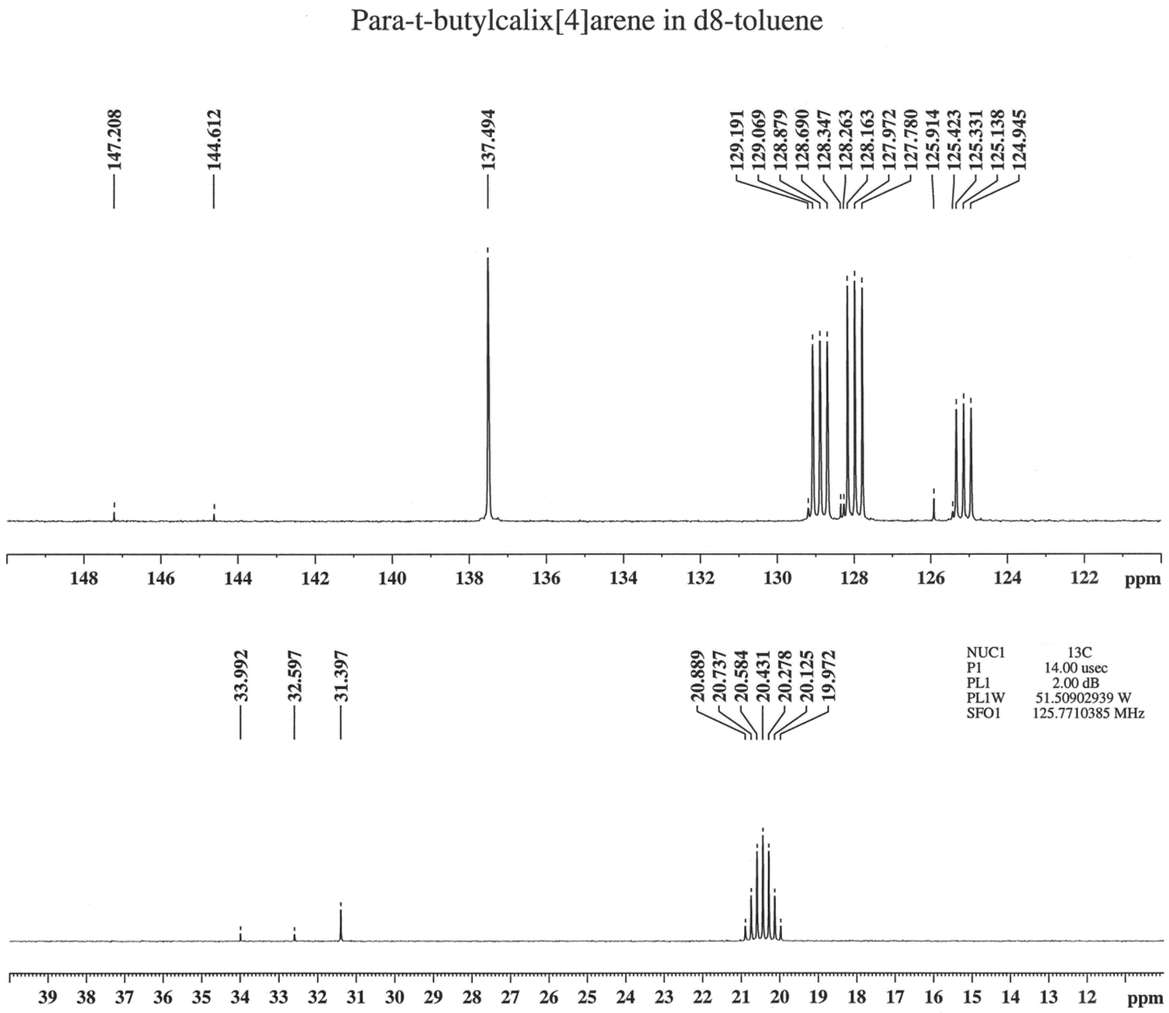

Supplementary Figure 23 | ^{13}C NMR spectra of *p*-*t*-butylcalix[4]arene. ^{13}C NMR spectrum (125 MHz, toluene-D_8) δ (ppm) 147.2, 144.6, 128.3, 125.3, 33.9, 32.6, 31.4.

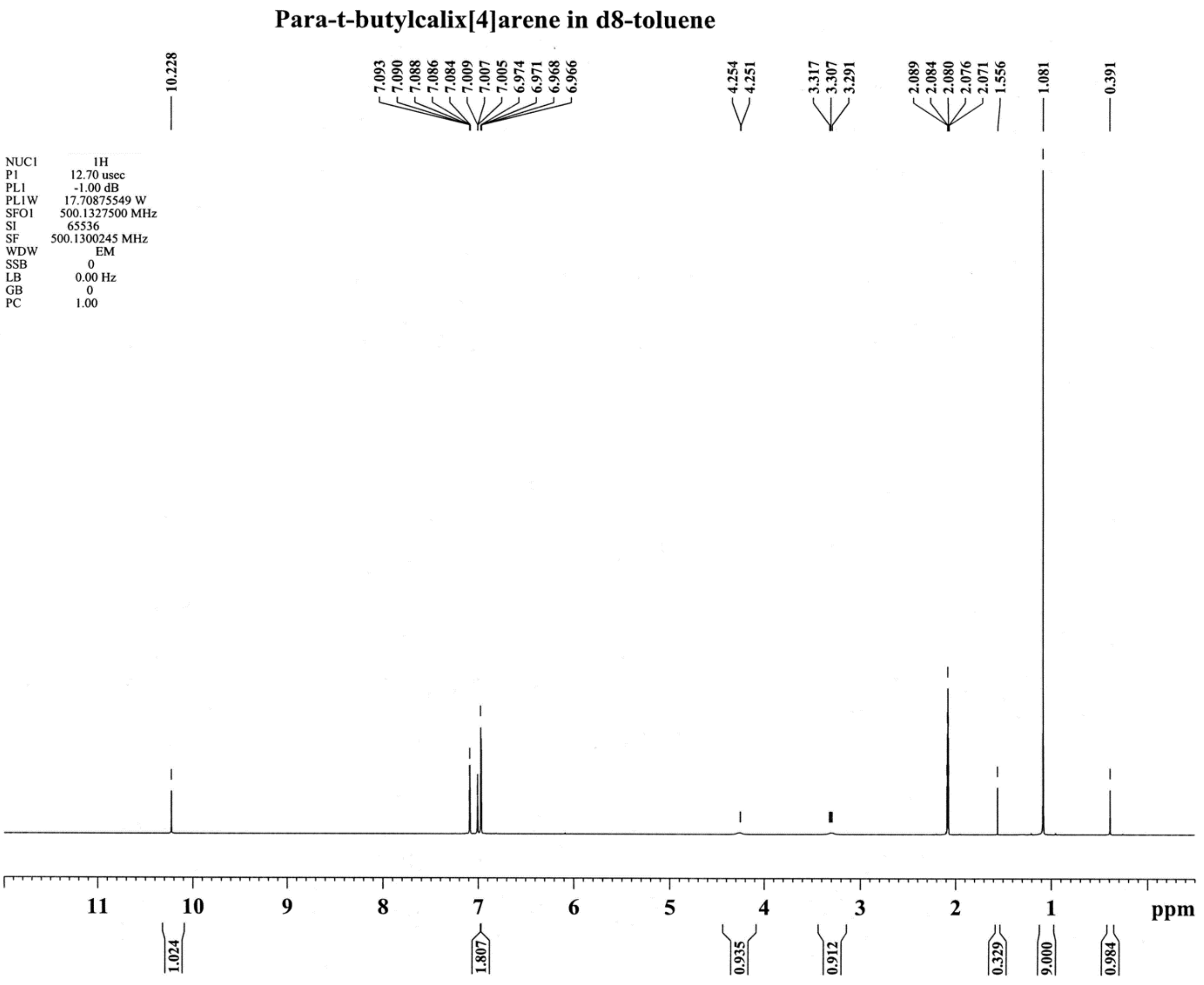

Supplementary Figure 24 | ¹H NMR spectra of *p*-*t*-butylcalix[4]arene. ¹H NMR spectrum (500 MHz, toluene-D_8) δ (ppm) 1.08 (s, 36H, -C(CH3)3), 3.3-4.2 (s, 8H, ArCH2Ar), 7.0 (s, 8H, Ar-H), 10.2 (s, 4H, -OH).

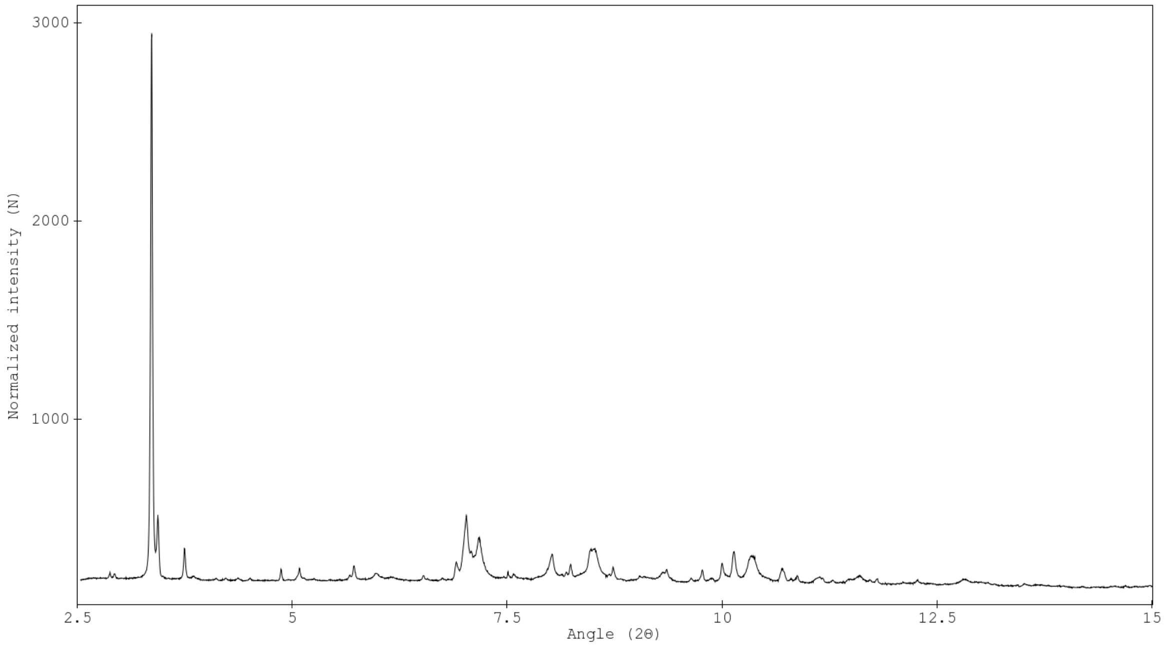

Supplementary Figure 25 | Synchrotron PXRD data of the sublimed *p*-*t*-butylcalix[4]arene. Data obtained at 293 K under vacuum with a X-ray wavelength of 0.77502 Å. High angle data were omitted for clarity purposes.

Supplementary Table 1 | Lattice constants and cation site occupancy of r2KCHA. In an atmosphere of vacuum and nitrogen, respectively, as a function of temperature determined by Rietveld refinement. (K1 denotes for potassium cation at site SII and K3 for site SIII' with a 2% error in occupancy).

Atmosphere	T (K)	lattice_a (Å)	lattice_c (Å)	volume (Å^3)	K1 occup.	K3 occup.	Rwp %	Romb_a	alpha
vacuum	150	13.4317	15.6946	2452.14	0.35	0.98	6.23	9.3577	91.7815
	170	13.4335	15.6861	2451.49	0.35	0.99	6.13	9.3568	91.8070
	190	13.4357	15.6769	2450.84	0.35	0.98	6.09	9.3566	91.8365
	213	13.4381	15.6668	2450.13	0.34	0.98	5.98	9.3559	91.8681
	233	13.4413	15.6578	2449.91	0.34	0.98	5.87	9.3559	91.9019
	253	13.4446	15.6459	2449.23	0.34	0.98	5.84	9.3556	91.9336
	263	13.4463	15.6401	2448.96	0.34	0.98	5.76	9.3554	91.9522
	273	13.4480	15.6337	2448.56	0.34	0.97	5.92	9.3550	91.9708
	283	13.4501	15.6261	2448.15	0.33	0.97	5.83	9.3549	91.9889
	293	13.4521	15.6203	2447.97	0.33	0.97	5.97	9.3548	92.0096
	303	13.4537	15.6155	2447.79	0.33	0.97	5.44	9.3545	92.0256
	313	13.4556	15.6105	2447.69	0.33	0.97	5.99	9.3546	92.0448
	323	13.4566	15.6044	2447.11	0.33	0.98	6.01	9.3541	92.0630
	333	13.4589	15.5995	2447.16	0.34	0.97	6.03	9.3543	92.0813
	363	13.4642	15.5823	2446.41	0.33	0.98	5.96	9.3539	92.1357
N_2	213	13.4623	15.6066	2449.51	0.38	1.00	6.06	9.3543	92.0644
	233	13.4641	15.5946	2448.31	0.38	0.99	6.07	9.3532	92.0996
	243	13.4652	15.5876	2447.60	0.37	0.99	6.17	9.3534	92.1211
	263	13.4680	15.5738	2446.44	0.37	0.99	6.06	9.3536	92.1613
	273	13.4692	15.5678	2445.95	0.37	0.99	6.16	9.3531	92.1805
	283	13.4705	15.5623	2445.56	0.38	0.99	6.19	9.3530	92.1973
	293	13.4726	15.5548	2445.14	0.38	0.99	6.34	9.3530	92.2152
	303	13.4737	15.5491	2444.63	0.37	0.98	6.47	9.3527	92.2315
	313	13.4754	15.5448	2444.59	0.37	0.99	6.44	9.3528	92.2480
	333	13.4783	15.5365	2444.33	0.37	0.99	6.37	9.3521	92.2820
	363	13.4834	15.5240	2444.22	0.36	0.97	6.35	9.3522	92.3311

Supplementary Table 2 | Diameter of the 8MR pore and distance between the pore-keeping cation and the pore framework as illustrated in Supplementary Fig. 2. Note the radii of the oxygen atom (1.35 Å) and potassium cation (1.33 Å) were subtracted to represent the effective distance available to a guest molecule.

temp (K)	8MR pore diameter in 3 directions (Å)			K^+ distance to oxygen in 8MR (Å)		
	D_1 (O1-O1)	D_2 (O2-O2)	D_3 (O3-O3)	K3-O1	K3-O2	K3-O3
150	3.079	4.281	4.558	0.24	0.979	0.841
170	3.077	4.281	4.543	0.239	0.972	0.841
190	3.085	4.278	4.513	0.243	0.957	0.839
213	3.087	4.279	4.512	0.244	0.956	0.839
233	3.092	4.277	4.481	0.246	0.94	0.839
253	3.1	4.277	4.469	0.25	0.935	0.838
263	3.11	4.274	4.467	0.255	0.933	0.837
273	3.107	4.273	4.453	0.253	0.926	0.837
283	3.103	4.273	4.436	0.252	0.918	0.836
293	3.104	4.267	4.429	0.252	0.914	0.833
303	3.108	4.266	4.417	0.254	0.908	0.833
313	3.11	4.265	4.391	0.255	0.895	0.833
323	3.121	4.267	4.391	0.261	0.896	0.834
333	3.116	4.265	4.375	0.258	0.887	0.833
363	3.125	4.261	4.336	0.263	0.868	0.831
393	3.146	4.274	4.228	0.273	0.837	0.814

Supplementary Table 3 | LJ force field parameters for the adsorbate-adsorbate interactions.

Adsorbate	mass	Interaction site	mass	ε (K)	σ (Å)
CH_4[3]	16.0	CH_4	16.00	158.5	3.72
N_2[4]	28.0	N	14.00	36.40	3.32
		N_{com}	0.000	0.000	0.00

Supplementary Table 4 | Buckingham parameters[5] for interaction of K with CHA framework along with charges used on framework atoms and on extra-framework K in parenthesis.

Cross species	Buckingham potential			Coulombic potential		
	**A** (eV)	**B (Å)**	**C** (eV)	**Charge (e)**		
K-O_z	5258.3	0.2916	193.7	K (0.99)	Si (2.21)	O_z^{Si}(-1.105)
					Al (2.08)	O_z^{Al}(-1.32)

Supplementary Table 5 | Force field parameters obtained for CH_4 and N_2 interactions with K-CHA.

Cross species	Fitted FF	
	**ε (K)**	**σ (Å)**
O_z-N	65.00	3.26
K-N	137.0	4.10
O_z-N_{com}	0.000	0.00
K-N_{com}	0.000	0.00
O-CH_4	130.0	3.75
K-CH_4	80.00	4.05

Supplementary Table 6 | x, y, z coordinates of the cavity centres in the r2KCHA supercell (3 × 3 × 3).

cavity #	x	y	z	cavity #	x	y	z
**1**	0.16049	0.16916	0.16687	**15**	0.50165	0.50127	0.50061
**2**	0.49382	0.16916	0.16687	**16**	0.16504	0.17395	0.50061
**3**	0.82715	0.16916	0.16687	**17**	0.8317	0.17395	0.50061
**4**	0.16652	0.49914	0.16687	**18**	0.49837	0.17395	0.50061
**5**	0.49985	0.49914	0.16687	**19**	0.16645	0.49985	0.83435
**6**	0.83318	0.49914	0.16687	**20**	0.83311	0.49985	0.83435
**7**	0.16191	0.82912	0.16687	**21**	0.49978	0.49985	0.83435
**8**	0.49524	0.82912	0.16687	**22**	0.83992	0.18228	0.83435
**9**	0.82857	0.82912	0.16687	**23**	0.17326	0.18228	0.83435
**10**	0.16646	0.83391	0.50061	**24**	0.50659	0.18228	0.83435
**11**	0.83312	0.83391	0.50061	**25**	0.83755	0.83857	0.8308
**12**	0.49979	0.83391	0.50061	**26**	0.50421	0.83857	0.8308
**13**	0.83498	0.50127	0.50061	**27**	0.17088	0.83857	0.8308
**14**	0.16832	0.50127	0.50061				

Supplementary Table 7 | Summary of best fit parameters of the LJM-Toth model. Underlined parameters are those constrained by independent experiments before model fitting.

Sorbent	Gas	Toth parameters				**LJM parameters**		Ext. surf.	Goodness of fit	
		n_∞ (mol kg^{-1})	B_0 (kPa^{-1})	ΔH (J mol^{-1})	m (-)	$\boldsymbol{T_0}$ **(K)**	$\boldsymbol{\beta}$ **(K)**	ε (-)	R^2	RMSE (mol kg^{-1})
**r2**KCHA	CH_4	2.093	8.65×10^{-7}	24.2×10^{3}	1	**266**	**16.5**	0.043[a]	0.981	0.0506
**r2**KCHA	N_2	1.661	1.49×10^{-6}	19.4×10^{3}	1	**254**	**18.6**	0.043[a]	0.992	0.0185
**r2**KCHA	Ar	2.602	6.10×10^{-5}	7.02×10^{3}	1	**245**	**38.3**	0.065	0.980	0.0112
**r2**KCHA	H_2	0.677	4.23×10^{-5}	6.71×10^{3}	1	**170**	**10.3**	0.14	0.986	4.42×10^{-3}
CX[4]	CH_4	1.125	5.53×10^{-6}	20.1×10^{3}	1	**230**	**62.7**	0	0.996	0.0187
CuTEI	CH_4	8.800	1.55×10^{-7}	29.5×10^{3}	0.150	**145**	**36.3**	0	0.961	0.0860

[a] Parameter ε calculated by S_{BET}(**r2**KCHA)/S_{BET}(**r5.5**KCHA) experimentally. **r5.5**KCHA is a potassium chabazite with Si/Al ratio of 5.5 whose BET surface area was measured to be 520 m^2 g^{-1}.

Supplementary Table 8 | The data points of CH_4 adsorption isotherms on CuTEI. Reproduced by digitizing the original curves at 273 K, 195 K, 179 K, 142 K and 113 K.[6] For isotherms at 179 and 142 K, only low pressure data points were collected for this study as the isotherms deviate from Langmurian shape at higher pressures possibly due to pore filling process.

T (K)	P (kPa)	n (mol kg^{-1})	T (K)	P (kPa)	n (mol kg^{-1})	T (K)	P (kPa)	n (mol kg^{-1})
273	2.2523	0.0081	195	35.6984	0.9183	179	11.3843	1.2162
273	5.3154	0.0190	195	40.7440	0.9564	179	13.7281	1.2735
273	8.7390	0.0352	195	43.5371	0.9727	179	15.8013	1.3198
273	10.0000	0.0399	195	46.6003	0.9890	179	18.2350	1.3743
273	13.1535	0.0515	195	49.4833	0.9998	179	21.3893	1.4233
273	17.1177	0.0705	195	52.6366	1.0133	142	0.5499	0.3355
273	20.0005	0.0759	195	55.6998	1.0296	142	1.1027	0.7774
273	22.7933	0.0840	195	59.0331	1.0377	142	2.0082	0.9465
273	25.4960	0.0948	195	61.8257	1.0404	142	3.0922	1.0528
273	33.9645	0.1191	195	64.7988	1.0512	142	4.4460	1.1455
273	45.2257	0.1489	195	68.2221	1.0566	142	8.8642	1.2954
273	57.9281	0.1785	195	71.9157	1.0647	142	10.0000	1.3204
273	78.5581	0.2162	195	74.8885	1.0673	142	10.8473	1.3390
273	86.3055	0.2269	195	78.4018	1.0700	142	12.8302	1.3771
273	96.7557	0.2457	195	81.3745	1.0699	142	15.0833	1.4153
273	107.5658	0.2537	195	85.1580	1.0725	142	17.5163	1.4425
195	0.5429	0.0845	195	89.7524	1.0779	142	20.3097	1.4724
195	1.2668	0.2018	195	94.0765	1.0805	142	23.5536	1.5051
195	3.0719	0.3245	179	0.1829	0.0982	113	0.9928	0.0682
195	5.1466	0.4254	179	0.5470	0.2318	113	3.3357	0.0954
195	7.4008	0.5044	179	0.7296	0.3191	113	8.3809	0.1143
195	9.8350	0.5726	179	1.0021	0.4009	113	10.0000	0.1194
195	10.0000	0.5765	179	1.7298	0.6546	113	12.7052	0.1279
195	12.3590	0.6325	179	2.3624	0.7255	113	15.8582	0.1305
195	15.1529	0.6815	179	3.0857	0.8209	113	22.3444	0.1385
195	18.0371	0.7360	179	4.0785	0.8891	113	40.7218	0.1599
195	21.4616	0.7850	179	5.1616	0.9655	113	46.3072	0.1679
195	24.7956	0.8204	179	6.1541	1.0200	113	57.9283	0.1840
195	27.6791	0.8503	179	7.6875	1.0936	113	85.4945	0.2160
195	30.2922	0.8748	179	9.4909	1.1536	113	106.5743	0.2319
195	33.1756	0.9020	179	10.0000	1.1704	113	110.2678	0.2372

Supplementary Table 9 | The quantities of hydrogen released from r2KCHA after dosing the zeolite with hydrogen at a pressure of approximately 100bar.

Repetition Number	Dosing Pressure (kPa±0.5kPa)	Amount of Hydrogen Released
1	10129.8	0.028mol±0.001mol
2	10336.0	0.0275mol±0.0009mol
3	10055.8	0.0271 mol±0.0009mol
4	10390.0	0.028 mol±0.001mol
5	10066.9	0.0272 mol±0.0009mol

Supplementary Note 1. Rietveld refinement of synchrotron PXRD for r2KCHA at different temperatures

Rietveld refinement was conducted to determine the lattice constants and the location of the atoms and pore-keeping cations of **r2**KCHA zeolite in the presence/absence of gas at all experimental temperatures. The results are shown in Supplementary Table 1 and the Crystallographic Information File (CIF) of the **r2**KCHA zeolite (provided as a separate file). The experimentally determined CIF files can be used to easily reconstruct the crystal structure of **r2**KCHA at any of the experimental temperatures. As clearly shown in Supplementary Table 1, the SIII' site at the centre of the 8MR pore was fully occupied across all temperatures under both vacuum and a N_2 atmosphere, and there was a very little change in unit cell constants suggesting the 8MR pores remain blocked by the K^+ cation.

To completely rule out the possibility that pore dilation governed the pore accessibility, we examined the diameter of the 8MR pore and the relative location of the pore keeping cation at the centre of the 8MR. As shown in Supplementary Fig. 2, we measured the distance between the framework oxygen atoms sitting opposite to each other in the 8MR, and the distance between the potassium cation at site SIII' and the surrounding oxygen atoms. The results are summarized in Supplementary Table 2. No significant change in the 8MR aperture size was observed based on the PXRD results spanning the temperature range 150 to 363 K. Specifically, the diameter of the 8MR in three different directions experienced a change of -0.02 Å, 0.046 Å, and -0.222 Å, respectively over this temperature range. At the same time, the K^+ cation was always at the centre of the 8MR pore aperture.

Supplementary Note 2. Rate of Adsorption Measurement

Section 5. Rate of adsorption study

Rate of adsorption measurements were carried out on **r2**KCHA with CH_4 as the adsorptive gas at different temperatures from 195 K to 333 K. The dynamic CH_4 uptake curves were recorded to show the adsorption capacity plateaued after a sufficiently long time. The rate of adsorption coefficient *k* was extracted from the curve based on the linear driving force model,[7] at 195 K, 279 K and 333 K representing the pore-inaccessible, transitional, and pore-accessible temperatures, respectively. The *k* values at 333 K and 279 K were found to be 7×10^{-3} s^{-1}, and 2.6×10^{-4} s^{-1} respectively, which is comparable to that of CH_4 on CMS (carbon molecular sieves) or zeolite 4A, indicating the thermodynamic equilibrium was reached.[8,9] The rate of adsorption measured at 195 K was actually greater than at the higher temperatures with a *k* of 2×10^{-2} s^{-1} In all three cases, the observed characteristic sorption times (1/*k*) are sufficiently short that the measured capacities could differ only slightly to those achieved after an arbitrarily long time. Furthermore, while the modest reduction in rate of adsorption resulting observed from 333 K to 279 K is expected from the standard theory of gas diffusivity, the increase in the observed sorption rate from 279 K to 195 K is not. This increase in sorption rate can be explained in terms the loss of access to internal adsorption sites and the rapidity with which external adsorption sites can be saturated. The fast rate of adsorption observed at 195 K then is characteristic of adsorption only onto the external surface sites and defects of the material. These results demonstrate that adsorption kinetics are not the determining factor governing the observed temperature-dependent pore accessibility.

Supplementary Note 3. Density functional theory (DFT) calculations

A rhombohedral lattice was used to construct the DFT model based on the Rietveld results from the synchrotron PXRD data. For all the DFT calculations, the cut-off energy of the plane wave basis-set was 405 eV. A gamma point only *k*-point mesh was used for one unit cell of chabazite (including three double six-ring prisms or one and a half supercavities). Such cut-off energy and *k*-point mesh have been tested to ensure the total energy value convergence within 1 meV/atom. The atomic positions were optimized with the conjugate gradient method until the forces acting on atoms were below 0.015 eV $Å^{-1}$, as suggested by Göltl and Hafner.[10] The DFT-D3 functional (with IVDW=11) was adopted to account for the van der Waals interactions, and the nudged-elastic-band (NEB) method for energy barrier calculations.

(1) Identification and justification of the pathway for cation deviation

We proposed that the door-keeping K cation has to move away from the 8MR aperture (site SIII') temporally and reversibly to allow for the admission of guest molecules, based on the fact that substantial gas adsorption occurs without permanent cation migration as observed in our *in situ* synchrotron PXRD of adsorption experiments (Fig. 1). We naturally considered that the K cation moves towards a secondary cation site, i.e., site II or site III[11,12] and determined using NEB calculations[13] the energy profile for both cation migration pathways under vacuum. The resulting cation migration profile suggests that the K cation would have to pass the saddle point to permit the entrance of any guest molecule in this study (H_2, N_2, Ar, or CH_4) if the cation were to follow the SIII'-to-SII pathway. This is because the opening of the pore will be not enough for the entrance of any molecule if the door-keeping K cation only reaches the saddle point (Supplementary Fig. 5) , given the K cation at saddle point along the SIII'-to-SII pathway is along the 8MR passage. A successful admission of a H_2 molecule necessitates the K cation relocation of at least 4.2 Å (diameter of H_2 plus the radius of K cation) along the SIII'-to-SII pathway. If this is the case, we should have observed a permanent cation migration to site SII from PXRD results; but we did not.

On the other hand, if the cation follows the SIII'-to-SIII pathway, the distance between K cation at site SIII' and at the saddle point is ~3.7 Å (Supplementary Fig. 6). If the K cation reaches the saddle point, it will be beyond the 8MR passage, and will give a completely unblocked doorway (distance between K cation at saddle point and the opposite oxygen of the 8MR is greater than 3.8 Å, larger than the guest molecules studied in this work). Thus, the entrance of the guest molecule is permitted without the K cation passing the saddle point, requesting no permanent cation migration to a secondary stable site. Therefore, we excluded the SIII'-to-SII pathway and adopted the SIII'-to-SIII pathway for further study.

(2) Calculation of the energy barrier for gas admission

The cation deviation and the gas entrance should occur simultaneously but the state of the art DFT calculations are not capable of treating such scenario straightforwardly. To overcome this difficulty, we adopted an iteration method with three steps:

Step one: we used NEB to determine the trajectory and corresponding energy profile for the cation moving from SIII' to the SIII **under vacuum**.

Step two: we used pseudo NEB to determine the energy profile for the cation movement following the trajectory determined in ***step one*** **in presence of different gases, respectively**. Thus, we determined a series of ΔE_{host} along the cation trajectory for each gas. Note that we did not only determine one ΔE_{host} at the saddle point because different gases may require the cation to move away (door open) to different extents before reaching the saddle point (door fully open). Therefore, for each gas, we plotted ΔE_{host} v.s. distance of cation movement (where the energy barrier value monotonically increases with distance till the saddle point since clearly the further the cation leaves away from SIII', the more energy toll paid by the cation).

Step three: we used NEB to determine ΔE_{guest} for each gas moving from inside the supercavity to the 8MR doorway **with the cation fixed at each location determined in *step two*, respectively**. We plotted ΔE_{guest} v.s. distance of cation movement (where the energy barrier value monotonically decreases with the distance since clearly the further the cation leaves away from SIII', the "wider opening" for the guest molecule and thus the less energy toll paid by the molecule).

ΔE_{total} for the admission of each gas was thus obtained by the summation of ΔE_{host} and the corresponding ΔE_{guest} at each point (Supplementary Fig. 7). By fitting the curve of ΔE_{total} vs distance of cation deviation and adopting the minimum, we determined ΔE_{total} for each gas.

Supplementary Note 4. Grand Canonical Monte Carlo (GCMC) simulations

(1) K-CHA Structure used

The experimentally determined lattice constants from PXRD for K-CHA, for example at 273K, are $a = b = c = 9.35504$ Å and $\alpha = \beta = \gamma = 91.9708°$. These lattice parameters remain almost constant in the temperature range used in this study according to the Rietveld synchrotron PXRD experimental data. Also, the experimental structures at various temperatures obtained do not distinguish between Al and Si atoms. Hence, the required number of Al atoms need to be generated by randomly replacing Si with Al in the CHA framework following the Lowenstein's rule which dismisses Al-O-Al linkage. Note that for such a high aluminium density chabazite like r2KCHA, the 8MR pore structure at least consists of one Al in any possible random placement of Al in the framework and the dwelling of the pore-keeping cation K^+ is ensured.

First, we generated a 3 × 3 × 3 supercell of the K-CHA framework and then replaced 101 Si atoms (out of 324) with Al atoms randomly to generate an aluminosilicate framework with Si:Al ~ 2.2. Then, 101 K ions (81 at SIII' and 20 at SII sites) were placed in the aluminosilicate framework. These K^+ cations may or may not be at equilibrated positions for the purpose of GCMC simulations and hence we used a parallel tempering procedure to equilibrate the K^+ positions. Obtaining good initial positions for the extra-framework cations (K^+) is essential to GCMC simulations. The details of the parallel tempering procedure can be found in the literature.[14-16] The single unit cell and the supercell of the CHA structure are shown in the Supplementary Fig. 8.

(2) Force fields used

2.1 Adsorbate-adsorbate interactions

For the adsorbate CH_4,[3] the spherical united atom model with zero net charge was used. This model uses only the dispersion interactions without any columbic interactions. For N_2, we used a 3-site model with two N atoms separated by a distance of 1.10 Å and a pseudo atom (N_{com}) at the centre of mass. The pseudo atom is used to create a quadrupole moment and can only be used with coulombic interactions and hence does not have dispersion interactions. The magnitude of the charge on each N atom is q_N= -0.482. A point charge of magnitude $-2q_N$ (+0.964) is located on a pseudo atom so that the total molecule charge is zero.[4] The dispersion interactions for CH_4-CH_4 and N_2-N_2 were defined using Lennard-Jones (LJ) 12-6 force fields (FF) from the literature as given in the Supplementary Table 3 with their respective references.

2.2 K^+-framework interactions

For K-framework interaction, we used the FF developed by Fang *et al.*[5] Here, K^+ as shown in Supplementary Fig. 8b interacts via two interactions: (i) Coulombic interactions with the framework atoms (Si, Al, O_z^{Si}, and O_z^{Al}) using the charges given in the Supplementary Table 4 where Si, Al, stand for Silicon and Aluminium while O_z^{Si} is oxygen connected to two Si, and O_z^{Al} is oxygen connected to Si and Al, and (ii) dispersion interactions (modelled here as a Buckingham potential instead of a LJ-12-6 type potential) between K and O_z (both $O_z^{Si}$and O_z^{Al}) atoms of a zeolite given by

$$E_{Buck}(R_{ij}) = A_{ij}\exp(-\frac{R_{ij}}{B_{ij}}) - \frac{C_{ij}}{R_{ij}^6} \qquad (1)$$

where, A_{ij}, B_{ij}, and C_{ij} are the Buckingham parameters for cross species *i* (K) and *j* (O_z) given in Supplementary Table 4 for K-O_z. The Buckingham terms for K-Si, K-Al, and K-K interactions are not explicitly considered, but taken into account through the effective potential with the oxygen atoms.[5]

2.3 Adsorbate-framework interactions

The force field parameters to model the interactions of CH_4 and N_2, with K-CHA in the GCMC simulations are not available in the literature. Hence, we developed our own force field for both the adsorbates with the K-CHA separately. The adsorbate-framework dispersion interactions were defined using Lennard-Jones (LJ) 12-6 force fields (FF). For columbic interactions, the charges used are already listed in the Supplementary Tables 3 and 4. In this work, we used our experimental data for the parametrization of the force field. Similar approaches have been used earlier,[3,17,18] where the force field parameters were fitted to adsorption isotherms data obtained from the experiments. The Lennard-Jones parameters of aluminium and silicon atoms with adsorbate molecules were excluded like many previous force fields.[3,18] This is because the polarizabilities of aluminium and silicon are lower than those of oxygen atoms resulting into shielding effect. Therefore, it is a reasonable assumption to ignore the LJ-12-6 terms for adsorbate-Si and adsorbate-Al interactions and thus include only adsorbate-O_z for simplicity.

First, we made an initial guess for the force field parameters for adsorbate-O_z ($O_z^{Si}$and O_z^{Al}) and adsorbate-K interactions by using the Lorentz-Berthelot mixing rule on adsorbate-adsorbate (from above section 2.1) and adsorbate-framework (CLAY FF for O_z-O_z and K-K)[19] interactions. Next, we selected a set of experimental adsorption isotherms at temperatures at which pores are not blocked by cations for the comparison. Then, we performed GCMC computations at five pressure points for each of the isotherms using the initial force field. We compared the calculated adsorption loading with the corresponding experimental data and if the deviation was large, then the LJ-12-6 (ε and σ) parameters were scaled one at a time for the adsorbate-O_z and adsorbate-K interactions till a reasonable fit of the adsorption isotherms was obtained. Also, while optimizing the parameters, care was taken to have a set of the force field parameters with reasonable physical meaning. In the initial stages of the iterative procedure, a coarse scaling of the LJ-12-6 parameters was done to make it computationally efficient, while in the later stages a fine scaling of the parameters was done to better fit the individual isotherms. The final parameters of the optimized FF are listed in the Supplementary Table 5.

(3) Blocking of the cavities

The number of cavities to be blocked at a given temperature for a given adsorbate was obtained from the percentage of accessible sites calculated from the regressed LJM-Langmuir models (see Fig. 4b). In the GCMC calculations, any blocked cavities were simply simulated by excluding a spherical volume around the cavity centre. The radius required to block a particular cavity completely is equal to the sum of the distance from the centre to the nearest framework atom (oxygen in this case) and the radius of that oxygen atom (~1.35 Å). The addition of 1.35 Å was needed because the actual CHA cavity is oval shaped and not actually spherical as assumed. After extensive calculations and visualization of the cavities, it was confirmed that this method will block the cavities perfectly without spilling out of the cavity and also no volume in the cavity is available for adsorption.

To find the centre of the cavity, the simulation box consisting of 27 unit cells (3 × 3 × 3) (Supplementary Fig. 9) was divided into discrete grid points with 0.2 Å spacing along each coordinate axis. Cavities, in which molecules can be adsorbed, were identified using an in-house code written in Fortran. In this code, for every grid point, the distance from the nearest framework atom was calculated.

The grid point corresponding to the maximum of these distances is one of the focal points of the approximately oval shaped cavity. Similarly, other focal point of the same cavity was obtained by finding the grid point which (i) is not the next nearest neighbour of the first focal point and (ii) has the next maximum distance from the framework atom. The midpoint between the two focal points is considered as the centre of the cavity. Remaining cavity centres were obtained by continuing the same process after excluding all the grid points present in the cavities already obtained. In the supercell, there are 27 cavities as shown by the coordinates of their centres in the Supplementary Table 6.

(4) GCMC simulation details and results

All the adsorption isotherms in this section were computed by the Grand Canonical Monte Carlo (GCMC) method using the RASPA software.[20,21] The LJ-12-6 potentials used were cut and shifted using a cut-off radius of 12 Å. Periodic boundary conditions were employed. Other GCMC simulation details are described in previous publications.[3,22] We used a 3 × 3 × 3 simulation box so that the minimum length in each of the coordinate directions was larger than 24 Å (which is double the cut-off radius). In all the simulations, 2×10^4 Monte Carlo cycles were used for equilibration and 1×10^5 Monte Carlo cycles were used for production. The Ewald summation method was used to calculate the electrostatic part of the interaction. K^+ cations were allowed to move during the GCMC simulations and could be wiggling around their starting positions.

First, we have generated GCMC predicted adsorption isotherms for N_2 and CH_4 in **r2**KCHA (Si:Al=2.2), at pressures between 0 to 110 kPa (see Supplementary Fig. 10(a) for N_2 and Supplementary Fig. 11(a) for CH_4) at various high temperatures with accessible pores for respective gases (above 283 K and 303 K for N_2 and CH_4 respectively). Not all of the isotherms are shown here for the clarity purposes. Firstly, the predicted adsorption isotherms match well with the experimental adsorption isotherms at higher temperatures at which pores are fully accessible. They also agree with low temperature isotherms extrapolated from LJM-Langmuir model assuming zero pore blocking. Secondly, we obtained the adsorption isotherms from GCMC at lower temperatures by blocking the corresponding number of pores determined from the LJM-Langmuir models. The predicted GCMC capacities were then found to be in excellent agreement with the experimental adsorption isotherms measured at lower temperatures, as shown in Supplementary Fig. 10(b) for N_2 and Supplementary Fig. 11(b) for CH_4.

Supplementary Note 5. Implementation of LJM models

(1) LJM for single component systems

The LJM-Toth model is given by substituting the standard Toth model into *Q(T,P)* in Equation (6):

$$n = [\Phi(1-\epsilon)+\epsilon]\,\frac{n_\infty b_0 exp\left(\frac{-\Delta H}{RT}\right)P}{\left\{1+\left[b_0 exp\left(\frac{-\Delta H}{RT}\right)P\right]^m\right\}^{\frac{1}{m}}} \tag{2}$$

Here n_∞ is the maximum monolayer adsorption capacity, b_0 the gas-solid affinity coefficient, ΔH the enthalpy of adsorption, R the gas constant, and m the surface heterogeneity coefficient. For homogeneous adsorption, m becomes unity and Supplementary Equation (2) reduces to the LJM-Langmuir model as follows:

$$n = [\Phi(1-\epsilon)+\epsilon]\,\frac{n_\infty b_0 exp\left(\frac{-\Delta H}{RT}\right)P}{1+b_0 exp\left(\frac{-\Delta H}{RT}\right)P} \tag{3}$$

The Levenberg–Marquardt method was used to find the best-fit parameters resulting from the regression of Supplementary Equations (2) and (3) to the experimental data. The goodness of fit is characterized by the square of correlation coefficient (R^2) and the root-mean-square-error (RMSE).

In the **r2**KCHA – gas system, we chose the LJM-Langmuir isotherm model to describe the complete experimental data sets, since adsorption isotherms of non-polar or inert gases on zeolites are normally of Langmuirian type. As demonstrated by the representative isobars shown in Fig. 3 and summarised in Supplementary Table 7, the LJM-Langmuir model achieved an excellent agreement of the experimental results for all four gases. The enthalpies of adsorption ΔH obtained for CH_4, N_2, Ar, and H_2 on **r2**KCHA (Supplementary Table 7), of 24.2, 19.4, 7.02, and 6.71 kJ mol^{-1}, respectively, are typical of similar adsorption on small pore zeolites. The fraction of external surface sites, ε, available for N_2 on **r2**KCHA was determined via independent experiments by using the 77 K N_2 BET surface area (S_{BET}) of **r2**KCHA (21 m^2 g^{-1}) divided by that of the closest non-trapdoor chabazite, i.e., **r5.5**KCHA (520 m^2 g^{-1}) which gave ε = 0.043. The second closest analogue is **r2**NaCHA (a sodium exchanged non-trapdoor chabazite)[23] with a S_{BET} of 490 m^2 g^{-1}. Using both values lead to very similar values of ε for **r2**KCHA. Given the size of a CH_4 molecule is quite close to that of N_2, the same ε was used for the analysis of CH_4 adsorption data as well. For the case of hydrogen or argon adsorption on **r2**KCHA, the external surface parameter ε was found by regression to the primary data set due to the lack of experimental data for "H_2 surface area" or "Ar surface area". We found a larger ε for H_2 (i.e., 0.14) compared with that estimated for N_2, suggesting hydrogen molecules with a much smaller size could access more external sites in the chabazite crystal facets and defects. Similarly, a value of ε = 0.065 was found for Ar which is in-between that of H_2 and N_2, and consistent with the order of their molecular sizes. Nevertheless, the model is relatively insensitive to ε and a variation of ε from 0.043 to 0.065 has an insignificant impact on the performance of the model.

It should be noted that the classical Toth/Langmuir parameters can be independently determined with a minimum of two adsorption isotherms if they were collected at temperatures well above T_0 (illustrated as Zone I in Supplementary Fig. 3). That means the two new parameters T_0 and β introduced by the LJM model are able to account for the transition behavior, i.e., the change of the pores from inaccessible to accessible (Zone II in Supplementary Fig. 3). For example, description of the eight N_2 isotherms from 223 K to 273 K would be completely impossible without the two LJM parameters. The complementary parameter ε accounts for the almost negligible uptake of the gases at temperatures far below the corresponding threshold admission temperature T_0 (Zone III in Supplementary Fig. 3).

(2) Extension of LJM model to multicomponent adsorption

It is a simple and straightforward method to apply the LJM model to the prediction of adsorption capacities of gas mixtures, because the multicomponent calculations require no experimental data for the mixture but only rely on LJM model parameters derived from pure component isotherm data. For example, to use the IAST model,[24] only the step in which spreading pressure is determined requires input from the parameters of the LJM model:

$$\int_0^{P_1^0} \frac{\mathrm{n}_1}{P_1} dP_1 = \int_0^{P_2^0} \frac{\mathrm{n}_2}{P_2} dP_2 = \cdots = \int_0^{P_i^0} \frac{\mathrm{n}_i}{P_i} dP_i \ \text{ for i} = 1,2,\dots,\mathrm{n} \quad (4)$$

Here the function of n_i represents the pure component adsorption isotherm characterized by an LJM model as described by Equation (6).

(3) Model determined T_0 vs $T(\mathrm{n_{max}})$

We also found the threshold temperature T_0 for gas admission does not align with the peak of the isobar, although the temperature at the peak, denoted as $T(\mathrm{n_{max}})$, was used as an arbitrary reference for the gas admission temperature in the past.[25] Comparing all the cases in this study, T_0 is found to be always lower than $T(\mathrm{n_{max}})$. On **r2**KCHA, this difference is around 10 – 15 K. Furthermore, unlike T_0, $T(\mathrm{n_{max}})$ is not a fixed value and varies with the isobar pressure. This is because the peak of the bell-shaped isobar is a trade-off between the LJM function for site accessibility (positive temperature derivative) and the Langmuir isotherm function for adsorption uptake (negative temperature derivative but positive pressure derivative) where higher pressures will result in an increase of $T(\mathrm{n_{max}})$. This is evident from the isobars observed for the trapdoor chabazite (Fig. 3) and *p*-*t*-butylcalix[4]arene (Fig. 5a). Therefore, we suggest that T_0 is a more appropriate parameter for characterizing the threshold admission temperature.

Supplementary Note 6. Procedures for H_2 storage, determination of amount of H_2 evolved and repeatability

(1) Experimental setup

The experimental setup and the procedure used for this experiment is described in Supplementary Fig. 20.

(2) Quantification of amount of hydrogen stored by encapsulation

The calculation of the amount of H_2 stored by encapsulation is presented as follows. Note that total amount of hydrogen stored (n_{recorded}) is the summation of three contributing terms: amount of desorbed (previously adsorbed on the internal surface of the zeolite), amount of decapsulated (molecules previously trapped in the zeolite intracrystal pore cavity), and residual amount (molecules re-adorbed after decapsulation). For the residual amount, it is a function of decapsulation pressure and temperature; if the release pressure is 1 bar and this term $n_{\text{resedual hydrogen}}$ is negligible.

$$n_{recorded} = n_{desorbed} + n_{decapsulated} + n_{residual\ hydrogen}$$

$$= \underbrace{m_{zeolite} \frac{n_{\infty,1} P_1}{\left(b + {P_1}^{m_1}\right)^{1/m_1}}}_{\text{Quantity of } H_2 \text{ adsorbed at critical encapsulation temperature and dosing pressure}} - \underbrace{m_{zeolite} \frac{n_{\infty,2} P_2}{\left(b_0 exp\left(\frac{Q}{RT_2}\right) + {P_2}^{m_2}\right)^{1/m_2}}}_{\text{Quantity of } H_2 \text{ adsorbed at released gas temperature and pressure}} + \underbrace{\frac{P_1 V_{pore\ space}}{T_1 R}}_{\text{Quantity of } H_2 \text{ in zeolite at dosing pressure}} - \underbrace{\frac{P_2 V_{pore\ space}}{T_2 R}}_{\text{Quantity of } H_2 \text{ in zeolite at released gas pressure}} + \underbrace{\frac{P_3 \left(V_{total} - V_{pore\ space}\right)}{T_{quench} R}}_{\text{Quantity of } H_2 \text{ in remaining apparatus at quenching temperature and atmospheric pressure}} \quad (5)$$

Supplementary Equation (5) was validated by comparing with the experimental data as shown by Supplementary Fig. 21. Noting that the storage capacity above 60 bar becomes linearly proportional to the initial dosing pressure, suggesting the term of n_{desorbed} flats off due to saturation on internal surface sites which is consistent with the weak adsorption of hydrogen on zeolites. The linearly increasing part of n_{recorded} is predominantly the contribution of $n_{\text{decapsulated}}$, which is proportional to the volume of internal pores of the chabzite available from literature. Therefore, the amount of hydrogen stored with much higher initial dosing pressures can be reasonably predicated using the knowledge of pore volume and the density of hydrogen at various temperature and pressures.

(3) Repeatability and reversibility test

For **r2**KCHA to be a suitable hydrogen storage material, it must be possible to repeat the encapsulation procedure multiple times with ease. The amount of hydrogen released from r2KCHA after dosing it with hydrogen at a pressure of approximately 100 bar on 5 separate occasions is given in Supplementary Table 9. The average amount of hydrogen released was found to be 4.65mol kg^{-1}, but more importantly the standard deviation of this value is just 0.07mol kg^{-1}. This is less than the standard error associated calculating the amount of hydrogen released. Although only five repetitions have been completed in this project, the initial findings are encouraging, in that there is no observed correlation between number of repetitions and the hydrogen storage capabilities of the zeolite. It should be noted that these repetitions were undertaken without sample reactivation. Many adsorbents that have reportedly stored large gravimetric densities of hydrogen require reactivation (e.g. metal hydrides), in that they must be heated to a substantially high temperature to remove all hydrogen before hydrogen can be stored at high gravimetric densities once more [26]. The relatively constant amount of hydrogen

released from **r2**KCHA after several repetitions of the encapsulation process means that the process is fully reversible, and as such this reactivation step is not required, giving the process a great advantage over other hydrogen storage mechanisms with comparable gravimetric densities of stored hydrogen.

Theoretically, adsorbents showing temperature-regulated admission for hydrogen can all be used for hydrogen storage without sustained pressure. Exploration of microporous materials with a higher threshold admission temperature T_0 for hydrogen is currently investigated. One promising strategy is to use Rb^+ or Cs^+ exchanged trapdoor chabazites as heavier cations are known to increase the threshold admission temperature.

(4) Conceptual tank-in-tank design for light weight on-board storage

We propose an on-board hydrogen (or methane) storage via repeatable encapsulation/decapsulation process. The mechanism of hydrogen filling, storage, use and re-fuelling is described in Supplementary Fig. 22. The on-board tank is low pressure rated to save the weight and it contains the to-be-improved trapdoor zeolite with a hydrogen threshold admission temperature between 50 and 100°C. While refuelling, the on-board tank is taken 'off-board', opened, and placed in a dosing tank designed to withstand very high pressures. The on-board tank is then warmed above the threshold admission temperature and dosed with high pressure hydrogen before being cooled below the threshold admission temperature to encapsulate the hydrogen. After the high pressure hydrogen in the free space is removed from the high pressure dosing tank, the on-board tank (now filled with encapsulated hydrogen at atmospheric pressure) can be closed and returned to its on-board location. At this point, as long as the on-board tank's temperature is kept below the hydrogen threshold admission temperature, atmospheric pressure will be maintained within the tank without any loss of hydrogen. Once the hydrogen is required for its on-board application, the on-board tank is carefully warmed, slowly releasing the hydrogen from the zeolite. Finally, when all the hydrogen originally encapsulated within the zeolite is used up, the tank is taken 'off-board' once more and the mechanism is repeated.

(5) CH_4 encapsulation in trapdoor zeolites and calix[4]arene, and ignition demonstration

In a similar setup, the encapsulation of methane was achieved at 195 K onto **r2**KCHA and *p*-*t*-butylcalix[4]arene with an initial doing pressure of 1 MPa and 2.2 MPa, respectively. We demonstrated a considerable amount of CH_4 gas can be stored without sustained pressure in a glass bottle at dry ice temperatures by encapsulation in **r2**KCHA trapdoor zeolites and the *p*-*t*-butylcalix[4]arene superamolecular hosts. When the bottle was placed in an ambient environment for around 5 minutes, CH_4 evolved gradually from the two materials, amounting to 7.4 ml g^{-1} and 25.9 ml g^{-1}, respectively. Interestingly, in the case of calixarene, the amount of CH_4 encapsulated equals approximately 1 CH_4 molecule for every two cages.

The methane was then ignited while being de-capsulated at ambient conditions for demonstration purposes (Supplementary Videos).

Supplementary References

1. Ma, S., Sun, D., Yuan, D., Wang, X.-S. & Zhou, H.-C. Preparation and gas adsorption studies of three mesh-adjustable molecular sieves with a common structure. *Journal of the American Chemical Society* **131**, 6445-6451, (2009).
2. Henke, S. & Fischer, R. A. Gated Channels in a Honeycomb-like Zinc−Dicarboxylate−Bipyridine Framework with Flexible Alkyl Ether Side Chains. *Journal of the American Chemical Society* **133**, 2064-2067, (2011).
3. Dubbeldam, D. *et al.* United Atom Force Field for Alkanes in Nanoporous Materials. *Journal of Physical Chemistry B* **108**, 12301-12313, (2004).
4. Murthy, C., Singer, K., Klein, M. & McDonald, I. Pairwise additive effective potentials for nitrogen. *Molecular Physics* **41**, 1387-1399, (1980).
5. Fang, H. *et al.* First principles derived, transferable force fields for CO_2 adsorption in Na-exchanged cationic zeolites. *Physical Chemistry Chemical Physics* **15**, 12882-12894, (2013).
6. Zhao, D., Yuan, D., Krishna, R., van Baten, J. M. & Zhou, H.-C. Thermosensitive gating effect and selective gas adsorption in a porous coordination nanocage. *Chemical Communications* **46**, 7352-7354, (2010).
7. Sircar, S. & Hufton, J. R. Why Does the Linear Driving Force Model for Adsorption Kinetics Work? *Adsorption* **6**, 137-147, (2000).
8. Xiao, G., Li, Z., Saleman, T. L. & May, E. F. Adsorption equilibria and kinetics of CH_4 and N_2 on commercial zeolites and carbons. *Adsorption*, 1-17, (2016).
9. Yang, Y., Ribeiro, A. M., Li, P., Yu, J.-G. & Rodrigues, A. E. Adsorption Equilibrium and Kinetics of Methane and Nitrogen on Carbon Molecular Sieve. *Ind. Eng. Chem. Res.* **53**, 16840-16850, (2014).
10. Göltl, F. & Hafner, J. Alkane adsorption in Na-exchanged chabazite: The influence of dispersion forces. *J. Chem. Phys.* **134**, 0641021-06410211, (2011).
11. Shang, J. *et al.* Potassium Chabazite: A Potential Nanocontainer for Gas Encapsulation. *J. Phys. Chem. C.* **114**, 22025-22031, (2010).
12. Shang, J. *et al.* Adsorption of CO_2, N_2, and CH_4 in Cs-exchanged chabazite: A combination of van der Waals density functional theory calculations and experiment study. *The Journal of Chemical Physics* **140**, 084705, (2014).
13. Henkelman, G., Uberuaga, B. P. & Jónsson, H. A climbing image nudged elastic band method for finding saddle points and minimum energy paths. *The Journal of Chemical Physics* **113**, 9901-9904, (2000).
14. Beauvais, C., Guerrault, X., Coudert, F. X., Boutin, A. & Fuchs, A. H. Distribution of sodium cations in faujasite-type zeolite: A canonical parallel tempering simulation study. *Journal of Physical Chemistry B* **108**, 399-404, (2004).
15. Earl, D. J. & Deem, M. W. Parallel tempering: Theory, applications, and new perspectives. *Physical Chemistry Chemical Physics* **7**, 3910-3916, (2005).
16. Fang, H. *et al.* Identification of High-CO_2-Capacity Cationic Zeolites by Accurate Computational Screening. *Chemistry of Materials* **28**, 3887-3896, (2016).
17. Jaramillo, E. & Chandross, M. Adsorption of small molecules in LTA zeolites. 1. NH_3, CO_2, and H_2O in zeolite 4A. *The Journal of Physical Chemistry B* **108**, 20155-20159, (2004).
18. García-Sánchez, A. *et al.* Transferable force field for carbon dioxide adsorption in zeolites. *The Journal of Physical Chemistry C* **113**, 8814-8820, (2009).
19. Cygan, R. T., Liang, J. J. & Kalinichev, A. G. Molecular Models of Hydroxide, Oxyhydroxide, and Clay Phases and the Development of a General Force Field. *Journal of Physical Chemistry B* **108**, 1255-1266, (2004).
20. Dubbeldam, D., Calero, S., Ellis, D. E. & Snurr, R. Q. RASPA: molecular simulation software for adsorption and diffusion in flexible nanoporous materials. *Molecular Simulation* **42**, 81-101, (2016).
21. Dubbeldam, D., Torres-Knoop, A. & Walton, K. S. On the inner workings of Monte Carlo codes. *Molecular Simulation* **39**, 1253-1292, (2013).
22. Calero, S. *et al.* Understanding the role of sodium during adsorption: A force field for alkanes in sodium-exchanged faujasites. *Journal of the American Chemical Society* **126**, 11377-11386, (2004).
23. Shang, J. *et al.* Determination of composition range for “molecular trapdoor” effect in chabazite zeolite. *The Journal of Physical Chemistry C* **117**, 12841-12847, (2013).

24. Tarafder, A. & Mazzotti, M. A Method for Deriving Explicit Binary Isotherms Obeying the Ideal Adsorbed Solution Theory. *Chemical Engineering & Technology* **35**, 102-108, (2012).
25. Shang, J. *et al.* Discriminative separation of gases by a "molecular trapdoor" mechanism in chabazite zeolites. *Journal of the American Chemical Society* **134**, 19246-19253, (2012).
26. Schlapbach, L. & Zuttel, A. Hydrogen-storage materials for mobile applications. *Nature* **414**, 353-358, (2001).
