# Supplementary material for: Temperature-regulated guest admission and release in microporous materials
Source: Nat Commun. 2017 Jun 9;8:15777. doi: 10.1038/ncomms15777 (PMC5472718; doi:10.1038/ncomms15777)
Supplement: Peer Review File [file ncomms15777-s4.pdf]

Editorial Note: this manuscript has been previously reviewed at another journal that is not operating a transparent peer review scheme. This document only contains reviewer comments and rebuttal letters for versions considered at Nature Communications.
